# Pseudouridines of tRNA Anticodon Stem-Loop Have Unexpected Role in Mutagenesis in *Pseudomonas* sp.

**DOI:** 10.3390/microorganisms9010025

**Published:** 2020-12-23

**Authors:** Mari Tagel, Heili Ilves, Margus Leppik, Karl Jürgenstein, Jaanus Remme, Maia Kivisaar

**Affiliations:** Institute of Molecular and Cell Biology, University of Tartu, 23 Riia Street, 51010 Tartu, Estonia; heili.ilves@ut.ee (H.I.); margus.leppik@ut.ee (M.L.); karl.jurgenstein@ut.ee (K.J.)

**Keywords:** TruA, RluA, pseudouridine, mutation frequency, *Pseudomonas putida*, *Pseudomonas aeruginosa*

## Abstract

Pseudouridines are known to be important for optimal translation. In this study we demonstrate an unexpected link between pseudouridylation of tRNA and mutation frequency in *Pseudomonas* species. We observed that the lack of pseudouridylation activity of pseudouridine synthases TruA or RluA elevates the mutation frequency in *Pseudomonas putida* 3 to 5-fold. The absence of TruA but not RluA elevates mutation frequency also in *Pseudomonas aeruginosa*. Based on the results of genetic studies and analysis of proteome data, the mutagenic effect of the pseudouridylation deficiency cannot be ascribed to the involvement of error-prone DNA polymerases or malfunctioning of DNA repair pathways. In addition, although the deficiency in TruA-dependent pseudouridylation made *P. putida* cells more sensitive to antimicrobial compounds that may cause oxidative stress and DNA damage, cultivation of bacteria in the presence of reactive oxygen species (ROS)-scavenging compounds did not eliminate the mutator phenotype. Thus, the elevated mutation frequency in the absence of tRNA pseudouridylation could be the result of a more specific response or, alternatively, of a cumulative effect of several small effects disturbing distinct cellular functions, which remain undetected when studied independently. This work suggests that pseudouridines link the translation machinery to mutation frequency.

## 1. Introduction

In addition to the four standard nucleotides, tRNA molecules acquire numerous post-transcriptional modifications, thus representing the most abundantly modified molecules in cell. Although some modifications have been proven to fulfil a role in tRNA structure formation and stabilization [1], translation decoding [2,3,4,5], maintenance of translation accuracy and processivity [6], as well as regulation of stress responses [7,8,9], the physiological role of several tRNA modifications is still enigmatic.

Pseudouridines (Ψs) are the most widespread nucleotide modifications. In bacteria the pseudouridylation is usually carried out by “stand-alone” enzymes that both recognize their RNA substrates and carry out the isomerization of uridine. Based on the protein sequence similarities, pseudouridine synthases are divided into five families. Synthases within one family show high conservation in protein sequences but protein sequence similarity between different families is low or totally absent [10,11]. Still, all known pseudouridine synthases possess several conserved structure motifs, similar tertiary structures and a catalytic aspartate residue, which is the only absolutely conserved amino acid essential for catalytic activity [12]. TruA and RluA are both prokaryotic pseudouridine synthases belonging to different families of Ψ synthases. Both enzymes modify U nucleotide(s) in the tRNA anticodon stem-loop (ASL).

TruA modifies U residues in the tRNA ASL, 3′ of the anticodon at positions 38, 39, and 40 [13,14]. Catalytically active TruA is a homodimer, and based on the *Thermus thermophilus* TruA crystal structure the tRNA-binding cleft is remarkably more flexible than in other Ψ synthases, allowing the same enzyme to modify uridines at positions 38–40 in tRNA [15]. Ψs at position 38–40 have been shown to have versatile role in different organisms. In general, it has been shown that Ψ at tRNA position 39 does not affect tRNA binding to the ribosome but improves its thermodynamic stability due to the nucleoside’s increased stacking ability [16,17]. In *Salmonella enterica* Typhimurium it was shown that Ψ38 and Ψ39 modifications stimulate the efficiency of ribosomal selection of leucine tRNAs, while Ψ40 did not have an effect on selection of the proline tRNA that carries this modification [18]. Moreover, it has been demonstrated that the lack of different modifications in the tRNA ASL increased +1 frameshift events, especially modifications at the positions 34 and 37. The lack of Ψ38 in tRNA^leu^_CUA_ decreased translation fidelity; however, it was rather specific for this particular tRNA since the lack of Ψ38–40 in other analyzed tRNAs did not affect translation fidelity [6]. The majority of studied tRNA modifications, including TruA modifications in *S. enterica* Typhimurium and *E. coli*, did not affect programmed −1 frameshifting [19]. Interestingly, in *Saccharomyces cerevisiae* pseudouridines at tRNA positions 38 and 39, synthesized by pseudouridine synthase Pus3p, affected growth in a temperature sensitive manner and exhibited decreased levels of stop codon readthrough and +1 frameshifting [20,21]. Also, in yeast there was a correlation between high −1 frameshifting efficiency and the presence of Ψ39 [22]. Thus, these modifications can have divergent effects on translation in different organisms, suggesting complex and context-dependent functions of tRNA modifications.

Regarding the other recorded phenotypes of TruA deficiency, it has been shown that in *S. enterica* Typhimurium the expression of *truA* is increased by H_2_O_2_ and the absence of *truA* reduces the virulence [23]. In *Pseudomonas aeruginosa truA* is required for the expression of type III secretion genes that are involved in pathogenesis [24]. Surprisingly, in *P. aeruginosa* the lack of *truA* does not affect cell growth [24], although in *E. coli* and in *S. enterica* Typhimurium its inactivation reduces growth remarkably [25].

RluA modifies tRNA ASLs 5′ of the anticodon, at position 32; the enzyme has dual-specificity, modifying also 23S rRNA at position 746 in addition to four tRNAs [26]. So far there are not many examples of modification enzymes being able to modify both tRNA and rRNA. In *E. coli* RluF pseudouridylates tRNA^tyr^ at anticodon position 35 and 23S rRNA at position 2604 [27] and RlmN methylates the C2 of adenines at position 37 in six different tRNAs as well as at position 2503 of 23S rRNA [28]. Ψ32 is also found in eukaryotes, e.g., in *S. cerevisiae* Rib2/Pus8p is responsible for Ψ32 in cytoplasmic tRNAs and Pus9p in mitochondrial tRNAs [29].

RluA recognizes a specific consensus sequence in an RNA loop-5′UUXXAAA-3′ (bases 32–38 in tRNA)—and modifies the first U [30]. It has been shown that an isolated ASL is a slightly less favored substrate for RluA than the full-length tRNA [31], which indicates that not only the nucleotide sequence but also the overall structure of the substrate affects the specificity of the enzyme. From the consensus sequence the most important nucleotides for RluA action seem to be at positions U33 and A36 [32]. It has been demonstrated that Ψ at position 32 has no effect on the ASL structure, but it makes the ASL thermodynamically more stable [33]. In general, nucleotides at tRNA positions 32 and 38 can establish hydrogen bonds which might be necessary for tRNA to form the correct U-turn [34] that is necessary to maintain the proper reading frame [35]. Overall, there are only few recorded *rluA* deletion phenotypes, e.g., in *E. coli* the lack of RluA did not alter growth of the strain; however, in growth competition experiments this strain was at a disadvantage compared to the parental wild-type [30].

Mutational processes are driving forces of evolution of bacteria. As most mutations are likely to be deleterious, the spontaneous mutation rate is generally held at a low level [36]. Nevertheless, growing evidence suggests that a variety of environmental stresses induce genomic changes in bacteria, thereby accelerating evolution of bacterial populations [37,38,39,40,41,42,43,44,45]. Mutation rates can also vary between different chromosomal regions [46,47,48,49]. The frequency of mutations can be elevated either constitutively, due to loss-of function mutations in genes for DNA repair systems [50], or transiently, in response to DNA damage or other stress situations [51,52,53,54,55,56]. Transiently increased mutability could be a consequence of the action of specialized DNA polymerases following exposure to DNA damaging agents or other environmental stresses [57,58]. Some recent studies have indicated that a network of factors affecting mutation frequency in bacteria could be much more sophisticated than initially presumed. For example, in addition to DNA replication and repair functions, the functionality of several transcription factors, enzymes participating in electron transfer chain and metabolic pathways [59] or affecting the redox status of the cell [60] may have a role in mutagenesis.

*Pseudomonas putida* is common in polluted soil and aquatic environments and is therefore extensively studied in respect of the stress tolerance mechanisms and adaptability [61,62]. In our pervious study we constructed a papillae-based test system which is based on levan production and can be used in a wide variety of *Pseudomonas* species to monitor mutation frequency in single colonies [63]. By implementing this test system in combination with transposon mutagenesis of *P. putida* we were able to find numerous potential genes affecting mutation frequency. The transposon insertion mutants which exhibited elevated mutation frequency in the papillae assay were also tested by using another assay that measured the frequency of spontaneous Rif^R^ mutants. To our surprise one of the biggest effects on mutation frequency appeared as a result of inactivation of the gene encoding pseudouridine synthase TruA.

In the present study we focused on functional studies of TruA and RluA in *P. putida* and demonstrated that inactivation of pseudouridylation activity of these enzymes increases mutation frequency in bacteria. TruA deficiency also elevated the mutation frequency in *P. aeruginosa*. The results of genetic studies and proteome analysis of *P. putida* TruA- and RluA-deficient mutants did not reveal any clear evidence that the mutator phenotype of these strains could be caused either by malfunctioning of common DNA repair pathways, intracellular ROS accumulation or upregulation of general stress response or SOS response.

## 2. Materials and Methods

### 2.1. Bacterial Strains, Plasmids, and Media

The bacterial strains and plasmids used in this study are listed in the Table S1. All *P. putida* strains are derivatives of PaW85 which is isogenic to KT2240, and *P. aeruginosa* strains are derivatives of PAO1-L. All *P. putida* strains were grown at temperature 30 °C, *E. coli* and *P. aeruginosa* strains were grown at 37 °C, except for measurement of Rif^R^ mutation frequency and stress tolerance assay, when the PAO1-L-derived strains were grown at 30 °C, just as *P. putida* strains. For complete medium either LB, YT or glc + CAA was used. For glc + CAA M9 buffer was supplemented with casamino acids (CAA) with tryptone and glucose both at final concentration 0.2%. Solid medium contained 1.5% Difco agar. Antibiotics were added at final concentrations: Kanamycin (Km) 50 µg·mL^−1^, gentamycin (Gm) 10 µg·mL^−1^, benzylpencillin (Bp) 1500–3000 µg·mL^−1^, rifampicin (Rif) 100 µg·mL^−1^. For *P. aeruginosa* Km was used at a concentration of 500 μg·mL^−l^ and carbenicillin (Cb) at a concentration of 400 μg·mL^−l^.

### 2.2. Construction of Plasmids and Strains

For all the single gene deletion strains and multiple gene deletion strains of *P. putida* and *P. aeruginosa* the method developed by Martínez-García and de Lorenzo was used [64]. For gene deletion approximately 500 bp DNA fragments from upstream and downstream of gene were amplified and fused into one ~1000 bp long fragment by overlap extension. The oligonucleotides used in this study are listed in the Table S3. The fusion PCR fragment was cut with restriction enzymes (BamHI, EcoRI, XbaI or SalI depending on fragment) and cloned into the corresponding sites of pEMG [64]. The cloning was carried out in *E. coli λpir* strain. Resulting suicide plasmids were electroporated into strains of interest and kanamycin-resistant colonies carrying a cointegrate in the chromosome were isolated on kanamycin selective plates. I-SceI nuclease was incorporated into cells in the composition of the expression plasmid Psw (I-SceI) [65] by electroporation. The nuclease was induced overnight in LB medium supplemented with 1.5 mM 3-methylbenzoate. Kanamycin-sensitive colonies were selected, and the deletion was verified by PCR. The pSW(I-SceI) plasmid was eliminated from cell by multiple inoculations into fresh medium and the absence of plasmid was verified with PCR.

For the complementation and overexpression studies the expression vector pSEVA/lacItac was generated. First the restriction sites for HindIII and XbaI were disrupted in the vector pSEVA-Km (RK2) and then the gene cassette *lacI-*P*_tac_* from pBRlacItac [66] was inserted as a BamHI fragment, resulting in the vector pSEVA/lacItac. In the case of *truA* or *rluA* complementation these genes were amplified by PCR from chromosome (Table S3) and inserted into the vector plasmid by using the HindII and SalI restriction sites, resulting in pSEVA/lacItactruA or pSEVA/lacItacrluA. The whole gene cassette from pSEVA was cloned into pGP-miniTn7-ΩGm [67] vector as a NotI fragment resulting in plasmids pGPTn7/truA and pGPTn7/rluA. All pervious steps were carried out in *E. coli*. For delivery of genes into Tn*7* insertion site in the chromosome of *P. putida* the published method was used [68]. *P. putida* strains lacking corresponding gene were co-electroporated with either plasmid pGPTn7/truA or pGPTn7/rluA and helper plasmid pUX-BF13 [69]. The cells were plated onto LB Gm plates and the insertion of locus was verified by PCR and sequencing (Table S3).

Catalytically inactive TruA or RluA were generated with two-step PCR. In first PCR step the *truA* sequence was amplified with primers Eco47-PPtruA and mut-PPtruAD70A and the *rluA* sequence with primers PPrluAsees and mut-PPrluAD57A. The primers mut-PPtruAD70A and mut-PPrluAD57A carried the mutated aspartic acid codon (mutated to alanine codon). For the second PCR step the previously generated PRC fragments were used as one primer and the second ones were complementary to the end of the gene. The generated chimeric PCR fragment should carry the mutation of interest. The PCR-amplified fragment was cut either with Eco47II (*truA*) or with PstI (*rluA*) and with SalI and was used to replace in either plasmid pSEVAKm/lacItactruA or pSEVAKm/lacItacrluA the original sequence cleaved with same enzymes. These manipulations resulted in vectors pSEVA/lacItactruA-mut and pSEVA/lacItacrluA-mut. Further steps were the same as described above for complementation of ΔtruA and ΔrluA stains with wild-type *truA* and *rluA* alleles. 

In the case of the construction of strains overexpressing PP1935, PP5487, or opr5487–89, the cloning procedure was similar to that of the construction of the *truA* and *rluA* complementation constructs. Amplified locus and pSEVA/lacItac were cleaved with PstI and XbaI and the gene cassette of interest was cloned into pBK-miniTn7-ΩGm [67] as a NotI fragment. Co-electroporation was carried out as previously described.

### 2.3. Virtual Analysis of tRNA Genes

All 75 tRNA genes of *P. putida* and 63 tRNA genes of *P. aeruginosa* were analyzed with the web tool tRNAscan-SE 2.0 [70] to predict their secondary structure and to identify in which positions near the anticodon U nucleotide is located.

### 2.4. tRNA Purification

*P. putida* overnight cultures were diluted 200 times into 400 mL of fresh 2YT medium and the cultures were grown for 5 h at 30 °C. Cells were collected by centrifugation and dissolved in 4 mL of TEN buffer (10 mM Tris pH = 7.5; 1 mM EDTA; 100 mM NaCl). Nucleic acid was extracted from the cells using 5 mL phenol pH = 7.6 treatment. Nucleic acid extraction was repeated for aqueous phase with equal volume of phenol pH = 5.0. Phenol was removed from aqueous phase with equal volume of chloroform. Nucleic acid was precipitated from aqueous phase with 2 volumes of ethanol and centrifugation. Nucleic acid was dissolved in 3 mL of GF buffer (20 mM Na-acetate; 1 mM EDTA and 100 mM NaCl). tRNA was purified using Superdex 200 column and ÄKTAPrime plus chromatography system (GE Healthcare, Chicago, IL, USA). GF buffer was used as gel filtration mobile phase. tRNA fraction was precipitated with 2 volumes of ethanol and centrifugation. tRNA was dissolved in MQ water. Agarose gel electrophoresis was used to assess the quality of purified tRNA population.

### 2.5. CMCT-Alkali Treatment

RNA CMCT-alkali treatment was done as described in Leppik et al., 2007 [71].

### 2.6. Primer Extension Analysis

The transcription from the *tac* promoter can be leaky even in the presence of repressor protein LacI [72,73]. Since we observed that the ability to pseudouridylate in TruA and RluA complementation strains was already restored without adding IPTG, the primer extension experiments with complementation strains were carried out without adding IPTG to avoid artificial overexpression of the genes. 1.5 µg of purified tRNA was mixed with 2 pmol of oligonucleotide in 1X AN buffer (50 mM K_HEPES pH = 7; 100 mM KCl) in 9 µL of reaction mixture. The tRNA was denatured at 90°C and temperature was slowly reduced to 45 °C for primer annealing. 1.2 µL of RB buffer (1.3 M Tris-HCl pH = 8.5; 100 mM MgCl_2_; 100 mM DTT), 0.8 µL dNTP(-C) MIX (110 µM dNTP but dCTP was 6 micromolar), 0.2 µL α^32^PdCTP (Hartmann Analytic) and 2 U of Reverse Transcriptase (Promega) was added to the reaction mixture in final volume of 12 µL. Primer extension labelling reaction was performed for 30 min at 42 °C. 2 µL of 1 mM dNTP was added to finish the primer extension reaction in 15 min at 42 °C. Nucleic acid was ethanol-precipitated from the reaction mixture and dissolved in formamide buffer. Primer extension reaction products were separated in 7% PAA gel and Amersham Typhoon scanner (GE Healthcare) was used to visualize radioactive isotope signal. 

### 2.7. Estimation of Spontaneous Mutation Frequency by Fluctuation Test

In order to estimate spontaneous mutation frequency, we performed the fluctuation tests and calculated the median value for mutants per 1 × 10^9^ cells as described in [74]. The frequency of Rif^R^ mutants was determined as described previously [75]. The cultures of *P. putida* and *P. aeruginosa* were grown into late-logarithmic growth phase in M9 medium containing glucose and CAA. To avoid pre-existing Rif^R^ mutants, cells were diluted 10^−5^ into fresh Glc + CAA minimal medium, dispensed into at least 10 test tubes as 2.3 mL aliquots and grown 20–22 h. If protein overexpression was needed, the corresponding genes were artificially overexpressed by adding 0.5 mM IPTG into growth medium. If effects of ROS on mutation frequency were examined, the growth medium was supplemented with 50 mM thiourea or 100 µM 2,2′-bipyridine. Approximately 5 × 10^8^ cells were plated from each independent culture onto LB plates containing 100 µg·mL^−1^ rifampicin, and for determination of colony forming units (CFU) in these cultures, cell dilutions were also plated onto LB plates without rifampicin. The Rif^R^ colonies were counted after 48 h of incubation at 30 °C. For every experiment the wild-type strain was included as a reference. At least 3 independent assays with 10 technical replicates were performed. 

### 2.8. Sequencing

DNA sequencing of the PCR products was performed by using the BigDye Terminator v3.1 Cycle Terminator kit (Thermo Fisher Scientific, Waltham, MA, USA) and analyzed with the Applied Biosystems 3730 × l DNA Sequencer. For Rif^R^ mutation spectrum from three different independent experiments random Rif^R^ mutants (one per plate) were selected and sequenced with primer PprpoB1 to verify the mutation in *rpoB* gene. 

### 2.9. Stress Tolerance Assay

To analyze different stressors and the stress tolerance, the cells were grown in glc + CAA media overnight and serially diluted bacterial cultures were spotted on LB agar plates supplemented with tetracycline (Tet, f.c for *P. putida* 2 µg·mL^−1^, f.c for *P. aeruginosa*), ampicillin (Amp, f.c. for *P. putida* 250 µg·mL^−1^, f.c for *P. aeruginosa*), and 4-nitroquinoline 1-oxide (NQO, f.c. 300 µM). Plates were incubated at 30 °C up to 48 h.

### 2.10. Proteome Analysis

For proteome analysis three independent cultures of each *P. putida* strain were grown overnight in glc + CAA media. Cells were diluted into fresh glc + CAA media to OD~0.1, cells were harvested in mid-log phased (OD580~1.0). Label-free quantification of whole cell proteome was performed by LC-MS/MS with LTQ-Orbitrap XL (Thermo Fisher Scientific) coupled to an Agilent 1200 nanoflow LC via nanoelectrospray ion source (Proxeon) in the Proteomics Core Facility, Institute of Technology, University of Tartu, Estonia. The data was analyzed using MaxQuant and Perseus software (Max Planck Institute of Biochemistry, Planegg, Germany) [76]. The whole dataset contained 3027 identified proteins. Parallel samples were grouped together, and groups were compared in pairs: (i) *P. putida* wild-type vs ΔtruA (2856 proteins); and (ii) *P. putida* wild-type vs ΔrluA (2842 proteins). To be included in the analysis, a protein needed to be detected in all three parallels of one group. Thereafter, missing values were imputed using default settings. Mean protein abundances were compared between two groups using the independent-sample Student t-test. The Benjamini–Hochberg multiple-testing correction was applied with the false discovery rate set to 0.05.

For on-off regulated proteins the initial dataset was analyzed without imputed values. These proteins that were present in all three samples of one strain and not in the samples of other strain were counted as on-off regulated proteins.

### 2.11. Statistical Analysis

The normality of dataset of interest was examined with Shapiro–Wilk W test. Since none of the results of fluctuation assays were normally distributed, the nonparametric analysis was used. The Kruskal–Wallis test was used for statistical analysis followed by Dunn’s post-hoc test. Calculations were performed using the Statistica 64 software (Tibco, Palo Alto, CA, USA).

### 2.12. Data Availability

The mass spectrometry proteomics data have been deposited to the ProteomeXchange Consortium via the PRIDE [77] partner repository with the dataset identifier PXD022353 and 10.6019/PXD022353. Supporting information for the proteome data presented in the table uploaded with the dataset PXD022353 is available in Supplementary Materials where this information is designated as Explanatory legend for Supplementary 2.

## 3. Results

### 3.1. In P. putida PaW85 TruA and RluA Target the Same Positions in tRNA as in E. coli 

**Figure 1:**
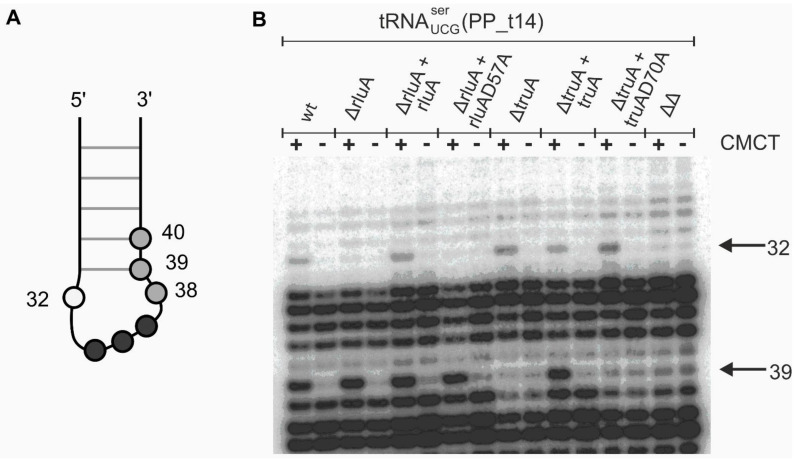
TruA and RluA specific pseudouridines in tRNA. (**A**) Schematic representation of tRNA anticodon stem loop (ALS) where black circles correspond to anticodon, white circle to RluA substrate position (32) and grey circles to TruA substrate positions (38-40). (**B**) Identification of TruA and RluA pseudouridylation sites in *Pseudomonas putida* PaW85 tRNA^ser^_UCG_ in wild-type (wt) and in *truA* and *rluA* deficient mutants by CMCT/alkali and primer extension analysis. Results of *truA* and *rluA* deletion strains (ΔtruA/ΔrluA), complementation strains with functional genes (ΔtruA + truA/ΔrluA + rluA), complementation strains with genes encoding catalytically inactive protein (ΔtruA + truA D70A/ΔrluA + rluA D57A) and *truA* and *rluA* double deletion strain (ΔΔ) are presented. The positions of Ψs were identified by transcriptase-directed primer extension stops specific to CMCT/alkali treatment with tRNA^ser^_UCG_-specific primer. “+” corresponds to the CMCT-treated lane and “−” to the untreated tRNA. The target positions of TruA (39) or RluA (32) in tRNA are indicated by arrow.

TruA and RluA catalyze isomerization of uridine (U) to pseudouridine (Ψ) in the anticodon stem-loop (ASL) of tRNAs (Figure 1A). They belong to pseudouridine synthase family I and III, respectively. Ψ synthases have a single catalytic Asp residue, Asp60 in *E. coli* TruA [78] and Asp64 in *E. coli* RluA [30], that is essential for the uridine isomerization reaction [79] at tRNA positions 38–40 and 32, respectively. Aligning TruA sequences of *E. coli* MG1655 and *P. putida* KT2440, which is isogenic to *P. putida* strain PaW85 used in the current study (Table S1), revealed 54.1% identity at the amino acid level (Figure S1). Asp70 of *P. putida* TruA represents the universally conserved and catalytically essential Asp residue of TruA enzymes (Asp60 in *E. coli*). Amino acid identity of *P. putida* and *E. coli* RluA is 48.8% (Figure S1), and the catalytic aspartate in *P. putida* resides at position 57 (Asp64 in *E. coli*). TruA modifies at least 17 tRNAs at positions 38–40 in *E. coli* [80,81]. According to in silico analysis, the *P. putida* KT2440 genome contains 75 tRNA genes, 43 of which are unique and of those, 19 tRNA species have one or more U nucleotides at TruA target positions—nucleotides 38, 39, and 40 of tRNA (see Table S2). Most potential TruA target tRNAs in *P. putida* overlap with *E. coli* TruA targets. In silico analysis indicated that the substrates for RluA in *P. putida* are almost the same as in *E. coli* [30,31]. Four tRNAs of *P. putida* carry the RluA consensus sequence 5′-UUXXAAA-3′, and all four overlap with potential TruA targets (see Table S2). RluA seems to modify 4 tRNAs in *E. coli* and *P. putida*, with the difference that *P. putida* RluA modifies one serine tRNA instead of two leucine tRNAs (Table S2).

To further confirm the substrates for TruA and RluA in *P. putida*, we performed primer extension analyses with 1-cyclohexyl-3-(2-morpholinoethyl)-carbodiimide metho-*p*-toluenesulfonate (CMCT)/alkali treated tRNAs of the *P. putida* wild-type strain and of the strains lacking TruA and/or RluA. Details for the construction of *truA* and *rluA* deletion strains and their complementation with wild-type or mutated *truA* and *rluA* genes are presented in Materials and Methods and in the Supplementary Materials (Tables S1 and S3). CMCT/alkali treatment causes primer extension stops at Ψ nucleotides. 5 potential TruA substrates (tRNA^leu^_CUG_, tRNA^leu^_UUG_, tRNA^cys^_UGC_, tRNA^tyr^_UAC_, and tRNA^ser^_UCG_) were subjected to primer extension analysis, and three of those were also potential substrates for RluA (tRNA^leu^_UUC_, tRNA^cys^_UGC_, and tRNA^ser^_UCG_). In Figure 1B the results for tRNA^ser^_UCG_ containing U nucleotides at the positions 39 and 32 are shown. We observed that in the *P. putida* wild-type strain these two U nucleotides were isomerized to Ψ as evidenced by primer extension stop in the CMCT treated samples. In contrast, in the strain lacking TruA (ΔtruA) there was no Ψ specific polymerase stop at position 39. Chromosomal complementation of the *truA* deletion strain with a functional *truA* gene (ΔtruA + truA) restored the primer extension stop at position 39, indicating that this site is pseudouridylated in the presence of the catalytically active TruA (Figure 1B). The Ψ specific signal was not detectable when the deletion of *truA* was complemented with an inactive TruA (ΔtruA + truA D70A) where the catalytic aspartate was mutated. These results confirmed that TruA catalyzes the formation of Ψ at position 39 in tRNA^ser^_UCG_ and that Asp70 is essential for this pseudouridylation reaction. TruA-directed pseudouridylation was also confirmed for the four other tRNAs analyzed (Figure S2).

The position 32 of tRNA^ser^_UCG_ remained unmodified when RluA was either missing (ΔrluA) or its catalytic Asp57 was mutated (ΔrluA + rluA D57A) (Figure 1B). Pseudouridylation at this position was restored when the deletion of *rluA* was complemented with functional RluA (ΔrluA + rluA) (Figure 1B). These results proved that *P. putida* RluA pseudouridylates uridine at the position 32 in tRNA^ser^_UCG_ and that Asp57 is essential for the catalytic activity. We also analyzed tRNA^leu^_UUG_ and tRNA^cys^_UGC_ and the same effect was observed (Figure S2). Analysis of the double mutant lacking both TruA and RluA (ΔΔ) confirmed our results demonstrating that there is no Ψ at position 39 or 32 in the absence of TruA and RluA (Figure 1B and Figure S2). Thus, both enzymes, TruA and RluA, have substrate specificities very similar to those of their *E. coli* counterparts.

### 3.2. Pseudouridines in the tRNA ASL Affect Mutation Frequency in P. putida

**Figure 2:**
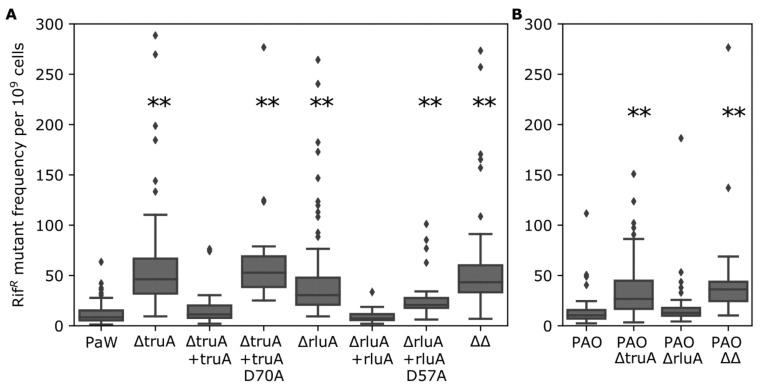
Comparison of Rif^R^ mutant frequencies of wild-type *P. putida* PaW85 and *truA* and *rluA* deletion strains (**A**) or wild-type *P. aeruginosa* PAO1-L and *truA* and *rluA* strains (**B**). The mean values (line in the box) of Rif^R^ mutant frequencies per 10^9^ cells are presented. The upper and lower borders of box represent third and first quartile, respectively, the whiskers represent non-outlier range and dimonds indicate outliers. In each strain *n* ≥ 40. ΔtruA + truA/ΔrluA + rluA—deletion strain complemented with functional gene; ΔtruA + truAD70A/ΔrluA + rluAD57A—deletion strain complemented with catalytically inactive TruA or RluA, respectively; ΔΔ—strain lacking both *truA* and *rluA*. “**” indicate *p*-value < 0.001 compared to the respective wild-type.

From our pervious genome-wide screen TruA was defined as a mutation frequency-affecting factor in *P. putida* [63]. To analyze the effect of TruA and RluA on mutation frequency in more detail, the frequency of the appearance of Rif^R^ mutants was measured in the wild-type *P. putida* PaW85 strain and in its Δ*truA* and Δ*rluA* derivative strains (Figure 2A). The absence of TruA caused an approximately 5-fold increase and the absence of RluA a 3-fold increase in the Rif^R^ mutant frequency. Both effects were statistically significant (Table S4). When the ΔtruA strain was complemented with the functional *truA* gene, the wild-type Rif^R^ mutant frequency was restored. The same effect was observed when the ΔrluA strain was complemented with functional RluA. In contrast, when the *truA* deletion was complemented with the catalytically inactive TruA (TruA D70A), the mutant frequency was comparable to that of the ΔtruA strain (Figure 2A). Similar results were obtained when the ΔrluA strain was complemented with the catalytically inactive RluA (RluA D57A) (Figure 2A). These results indicated that the mutant frequency is elevated due to the absence of catalytic activity of these tRNA modification enzymes. Our results also revealed that the strain lacking both TruA and RluA had no cumulative effect; the mutant frequency in the double mutant was comparable with that of the strain lacking only TruA (Figure 2A). Although the *truA* and *rluA* genes were incorporated in the complementation studies into the chromosome under the control of P*_tac_* promoter and its repressor LacI, the initial phenotype was already restored without IPTG, the adding of IPTG had no additional effect on Rif^R^ mutant frequency. This implied that the leaky transcription of the *truA* or *rluA* genes from the P*_tac_* promoter was sufficient to restore the wild-type phenotype (Figure S3). Here it is important to note that the leakiness of the P*_tac_* promoter is a known phenomenon; this has been previously documented in our laboratory and also reported by other research groups (see, e.g., [72,73]). Taking together, the results of the current study demonstrate that the absence of pseudouridylation in the ASL of tRNAs by TruA or RluA elevates Rif^R^ mutant frequency in *P. putida*.

To investigate whether the lack of TruA or RluA activity on mutation frequency is not restricted only to *P. putida*, we also constructed *truA* and *rluA* deletion strains of *P. aeruginosa* PAO1-L. Comparison of the Rif^R^ mutant frequency in the *P. aeruginosa* wild-type strain and its derivatives lacking TruA and/or RluA revealed that the absence of TruA elevated mutation frequency significantly also in *P. aeruginosa*; however, the observed effect was lower than in *P. putida* and the absence of RluA had no significant effect on Rif^R^ mutant frequency in *P. aeruginosa* (Figure 2B, Table S5). As in *P. putida*, the mutant frequency of the *P. aeruginosa* double mutant was comparable to the mutant frequency measured in the ΔtruA strain. 

It has been shown that tRNA modifications can be important for maintaining translation fidelity [6], and in some cases mutants with altered translation fidelity can achieve phenotypic resistance to rifampicin without any changes in the *rpoB* sequence [82]. Therefore, to confirm that the observed Rif^R^ colonies represent true mutants, we sequenced the *rpoB* gene in about 60 randomly selected *P. putida* Rif^R^ colonies that emerged in independent cultures of the ΔtruA and ΔrluA *P. putida* strains. All the sequenced mutants had a mutation in the *rpoB* gene (Table S6). Differences in the spectrum of mutations could hint to differences in DNA replication fidelity and/or malfunction of certain DNA repair pathway(s). However, analysis of the spectrum of Rif^R^ mutations in the ΔtruA and ΔrluA strains did not reveal any specific pattern in comparison with that of the wild-type strain (Table S6).

### 3.3. Elevated Rif^R^ Mutant Frequency in the Strains Lacking TruA or RluA is Not the Result of Malfunction of Major DNA Repair Pathways in P. putida

**Figure 3:**
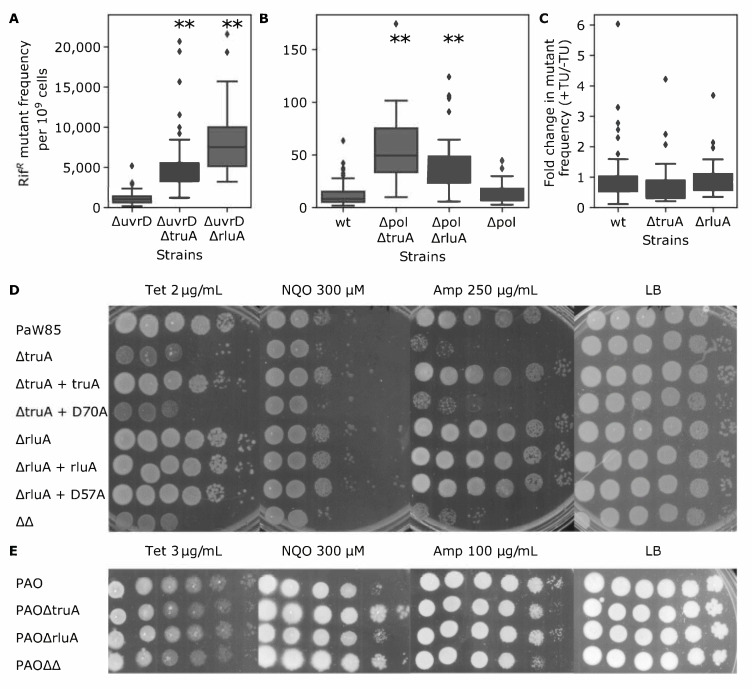
Comparison of Rif^R^ mutant frequencies in TruA and RluA proficient and deficient strains lacking additionally DNA repair or TLS polymerases functions, and estimation of phenotypic effects of the absence of TruA and RluA on stress tolerance. The mean values (line in the box) of Rif^R^ mutant frequencies per 10^9^ live cells are presented. The upper and lower borders of box represent third and first quartile, respectively, the whiskers represent non-outlier range and diamonds correspond to outliers. In each strain *n* ≥ 30. (**A**) *uvrD* and *truA* or *rluA* double mutant compared to the *uvrD* mutant strain, “**” indicate *p*-value <0.001 compared to ΔuvrD. (**B**) *P. putida* PaW85 (wt) compared to TLS DNA polymerases mutant (Δpol) and Δpol lacking *truA* or *rluA*. Δpol—*P. putida* strain lacking *imuAB*, *imuC*, *polB,* and *dinB* genes, “**” indicate *p*-value <0.001 compared to the wild-type strain. (**C**) Mutant frequency change of the wild-type *P. putida* PaW85 (wt), *truA* deletion strain (ΔtruA) and *rluA* deletion strain (ΔrluA) with (+) and without (−) thiourea (TU). The ratio of Rif^R^ mutant frequency with TU and without TU is shown. The mean values (line in the box) of ratio are presented. The upper and lower borders of the box represent third and first quartile, respectively, the whiskers represent non-outlier range and dimonds indicate outliers. In each strain *n* = 30. Panels (**D**,**E**) show plate assay of the stress tolerance of the *P. putida* PaW85 wild-type strain and the *truA* and *rluA* deficient strains and the *P. aeruginosa* PAO1-L wild-type strain and the *truA* and *rluA* deficient strains, respectively. Tenfold dilutions of overnight culture were spotted onto LB agar plates containing indicated chemical and incubated at 30 °C for 24 h (Tet, 48 h) for *P. putida* and 48 h for *P. aeruginosa*. ΔtruA + truA/ΔrluA + rluA—deletion strain complemented with functional gene; ΔtruA + truAD70A/ΔrluA + rluAD57A—deletion strain complemented with catalytically inactive TruA or RluA, respectively; ΔΔ—strain lacking both *truA* and *rluA*.

To analyze whether the malfunctioning of DNA repair pathways might have caused the observed mutator phenotypes, we monitored the Rif^R^ mutant frequency in the TruA- and RluA-deficient strains in the absence of UvrD. UvrD is essential in the DNA mismatch repair (MMR) and nucleotide excision repair (NER) pathways. If the elevated mutant frequency in ΔtruA and ΔrluA strains is caused by malfunctioning of MMR or NER repair pathways, no differences between the *uvrD* deletion strain and the *truA uvrD* or *rluA uvrD* double mutant are expected. However, the results presented in Figure 3A revealed that although cells without UvrD had remarkably higher mutant frequency than the wild-type *P. putida* PaW85 (Figure 3B), the *uvrD* deletion in the ΔtruA and ΔrulA strains resulted in statistically higher mutant frequencies compared to the *uvrD* single mutant (Figure 3A, Table S7). These results indicated that the elevated Rif^R^ mutant frequency in the ΔtruA and ΔrluA strains is not caused by the malfunctioning of the MMR or NER pathway. Unexpectedly, the combined effect of the deletion *uvrD* and *rluA* was higher than that of the deletion of *uvrD* and *truA*. Whether there is some kind of backup system for UvrD function, which is more severely impaired in the absence of RluA, needs further investigations.

Another factor which could cause elevated mutation frequency in bacteria is the SOS response. The SOS response elevates mutation frequency mainly by upregulation of “error-prone” specialized DNA polymerases [57]. In *E. coli*, after induction of the SOS response, three out of five DNA polymerases are upregulated: Pol II (*polB*), Pol IV (*dinB*), and Pol V (*umuDC*) [83]. In *P. putida* there is no Pol V and the SOS response differs from that of *E. coli*, involving two LexA regulators and theDNA damage-inducible *imuABC* cassette [84,85]. We hypothesized that the absence of TruA and RluA could cause activation of the SOS response in *P. putida*, which in turn may lead to the “error-prone” DNA synthesis, thereby elevating Rif^R^ mutant frequency. If this is the case, then we should not observe increases in mutant frequency in the ΔtruA and ΔrluA strains in the absence of these DNA polymerases. To test this hypothesis, we constructed Δpol strain lacking *polB* (Pol II), *dinB* (Pol IV), and the mutagenic operon (*imuABC*) where *imuC* (known also as *dnaE2*) codes for a DNA polymerase that is homologous to the α-subunit of the replicative DNA polymerase. The results presented in Figure 3B revealed that the Rif^R^ mutant frequencies were comparable between the Δpol and the wild-type strain. Deleting the *truA* or *rluA* genes in the Δpol strain resulted in mutant frequencies approximately 5- and 3-fold higher, respectively, than measured in the parental wild-type or Δpol strain (Figure 3B, Table S8). These results implied that the elevated Rif^R^ mutant frequency in the *truA* and *rluA* deletion strains is not caused by the action of the studied DNA polymerases. 

### 3.4. Addition of ROS Scavenging Agents Does not Affect the Rif^R^ Mutant Frequency of the P. putida ΔtruA and ΔrluA Strains

The metabolic versatility of *P. putida* indicates that this bacterium is able to adapt to diverse environments with different stressors. The most common exogenous and endogenous stressors are reactive oxygen species (ROS). It has been shown that some tRNA modifications (e.g., methylation and thiolation) can modulate oxidative stress response in bacteria [8,9,86]. Adding ROS scavenging agents to the growth medium of bacteria could indicate whether the elevated mutation frequency originates from reduced tolerance to intracellular ROS levels [87]. To analyze if the elevated Rif^R^ mutant frequency of ΔtruA and ΔrluA strains in *P. putida* might originate from increased levels of intracellular ROS or reduced ROS tolerance, the ROS scavenging agent thiourea (TU) was added to exponentially growing cell cultures at a final concentration of 50 mM, and the ratio of mutant frequency with and without TU was compared. We expected that if the intracellular level of ROS is increased and the elevated mutation frequency is caused by ROS, the addition of a reducing agent could decrease the amount of ROS and reduce the Rif^R^ mutant frequency in the TruA and RluA-deficient strains. However, we did not observe any statistically significant reduction in the Rif^R^ mutant frequency when TU was added to the growth medium of the *P. putida truA* and *rluA* defective strains (Figure 3C, Table S9). These results implied that the elevated mutation frequency in these strains is not caused by increased intracellular ROS levels. 

### 3.5. The Absence of TruA-Dependent Ψs Decreases Stress Tolerance of P. putida

Although the results of the above-described assays with ROS scavenging chemicals (Figure 3C) did not support the idea that the elevated mutation frequency in the absence of tRNA pseudouridylation could be associated with the increased amount of ROS in cells, these negative results did not exclude the possibility that the lack of Ψs in ASL of tRNAs makes bacteria more susceptible to various stressors, which in turn could affect mutation frequency. To compare stress tolerance of the *P. putida* wild-type strain and its ΔtruA and ΔrluA derivatives lacking pseudouridines at specific positions of the tRNA ASL, bacteria were grown overnight in glc + CAA medium and the cultures were diluted onto LB plates containing various stressors. The effect of chemicals that affect translation (tetracycline), cell wall synthesis (ampicillin) or induce ROS production (4-Nitroquinoline 1-oxide, NQO) was tested. We observed that in the case of the strains either lacking or containing a nonfunctional TruA, tetracycline, ampicillin and NQO caused growth inhibition (Figure 3D). The complementation of the ΔtruA strain with the functional TruA but not with TruAD70A restored the wild-type phenotype (Figure 3D). Therefore, it can be concluded that the functional TruA and its pseudouridylation activity is important for the stress tolerance in the case of the aforementioned stressors. It should be noted that the absence of TruA did not decrease the viability of *P. putida* on LB medium lacking these chemicals. In contrast to TruA, there was no change in the viability of bacteria either lacking RluA or carrying its nonfunctional derivative in the presence of the studied chemicals (Figure 3D). 

In addition to *P. putida*, we also analyzed the stress tolerance of *P. aeruginosa* ΔtruA and ΔrluA strains in the presence of the same chemicals. Different from the corresponding *P. putida* strain*,* no significant effect on the viability of *P. aeruginosa* lacking TruA was observed in the presence of tetracycline and ampicillin (Figure 3E). In the case of NQO, surprisingly, the strains lacking TruA tolerated the given stress conditions even better than the wild-type strain (Figure 3E). This was opposite to the NQO effect observed in *P. putida*. Like in *P. putida*, the lack of RluA did not have any effect on the stress tolerance in *P. aeruginosa*, except for ampicillin treatment, conditions under which the *truA*/*rluA* double mutant showed slightly reduced growth in comparison with the wild-type strain (Figure 3E).

### 3.6. Proteome Analysis Revealed A Wide Spectrum of Changes in the Absence of TruA But Minor Changes in the Absence of RluA

**Figure 4:**
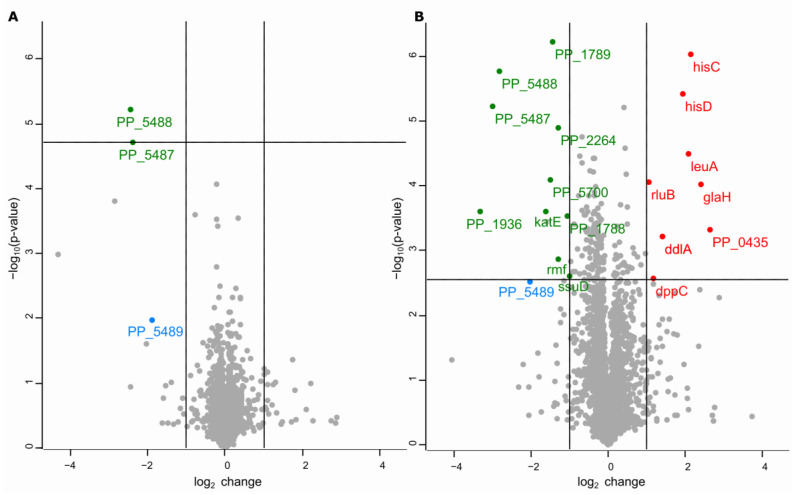
Overview of *P. putida* wild-type and *rluA* (**A**) or *truA* (**B**) mutant full proteome comparison. In volcano plot every dot represents a protein. The horizontal line indicates the statistical significance threshold after Benjamin–Hochberg multiple testing correction [false discovery rate (FDR) = 0.05]. The vertical lines indicate twofold difference between compared proteomes. Statistically significant at least two-fold increase in protein level are presented as red dots with the gene name and at least two-fold decrease in protein level are presented as green dots. Blue dot represents the third protein of PP5487-PP5489 operon.

To gain insight into cellular responses in the absence of TruA or RluA, we performed whole proteome analysis of mid-log phase cells of *P. putida* using quantitative mass-spectrometry and label-free quantification. Data are available via ProteomeXchange with the identifier PXD022353. Comparison of the full proteome of the *rluA* mutant versus the wild-type *P. putida* revealed rather small changes of protein expression levels. After applying multiple testing corrections to 2842 proteins quantified in both strains, the downregulation of only two uncharacterized proteins encoded by *PP5487* and *PP5488* that belong to the same operon was statistically significant (Figure 4A, Table 1). The third protein PP5489 encoded by this operon was downregulated, but the change was not statistically significant (Figure 4A). 

Differences between the wild-type and ΔtruA strain proteomes were more apparent. Out of 2856 proteins quantified the expression change of 158 proteins was statistically significant and out of those 18 proteins differed at least twofold (Figure 4B, Table 1). Most of these proteins were functionally unrelated. Ten of those proteins were downregulated and 8 upregulated. For example, some proteins participating in different amino acid biosynthesis pathways were upregulated (HisD, HisC, LeuA), and several stress-regulated proteins were up- (CsiD) or downregulated (catalase KatE, Rmf) (Table 1). The results of the proteome comparisons are consistent with the previously described results, indicating that ΔtruA contrary to ΔrluA, has several easily detectable phenotypes, which is in line with the protein expression pattern of the ΔrluA strain largely resembling that of the wild-type strain. It is also noteworthy that none of the proteins known to participate in DNA replication or DNA repair, including DnaQ, a protein whose activity is responsible for the proofreading activity of the replicative DNA polymerase Pol III, showed evidence for expression changes in the ΔtruA and ΔrluA strains in comparison with the wild-type (see table in the dataset PXD022353).

Interestingly, the operon *PP5487-89* that was downregulated in the ΔrluA strain, was also downregulated in the ΔtruA strain, and the downregulation of the proteins corresponding to the first two genes of the operon was statistically significant (Figure 4, Table 1). In addition, in both strains there were so-called on-off regulated proteins (proteins that were detected in all three samples of one strain but not in the samples of the other strain). We detected three on-off regulated proteins in the comparison of the wild-type strain with the *rluA* mutant and five with the *truA* mutant (Table 1). Among these proteins one putative transcriptional regulator, PP1935, was present in all independent samples of the ΔtruA and ΔrluA strains, but was not detectable in the samples of the wild-type strain. Interestingly, the gene of this transcriptional regulator is located just downstream of the aforementioned operon *PP5487-PP5489* and is transcribed in the opposite direction, which makes it possible that PP1935 could downregulate the transcription of the operon *PP5487-89*. It should be noted that all these genes (Table 1) are located in a large (about ~65 kb-long) genomic island which is of phage origin. This prophage appears to be *P. putida* KT2440-specific, since the other sequenced *P. putida* strains do not harbour it [88]. The proteome data also revealed that changes in the expression level of these particular proteins is rather specific, as the expression of other proteins encoded by the same prophage was not changed when TruA or RluA were absent.

### 3.7. The Mutator Phenotype of P. putida ΔtruA and ΔrluA Strains Is Not the Outcome of Downregulation of the Operon PP5487-PP5489

Since downregulation of hypothetical proteins encoded by the *PP5487-PP5489* operon and increased expression of the *PP1935-*encoded putative transcriptional regulator were the only predominant changes simultaneously observed in ΔtruA and ΔrluA proteomes, our next experiments were focused on examining the possibility that changes in the expression level of these proteins could be connected with changes in mutation frequency in *P. putida* cells. For this purpose, we constructed strains either overexpressing the first gene of the operon (tacPP5487), the whole operon (tacPP5487-89), or the putative transcriptional regulator (tacPP1935), and also strains either deleted for the first gene of the operon (ΔPP5487), or the gene of the putative transcriptional regulator (ΔPP1935) (see Materials and Methods).

**Figure 5:**
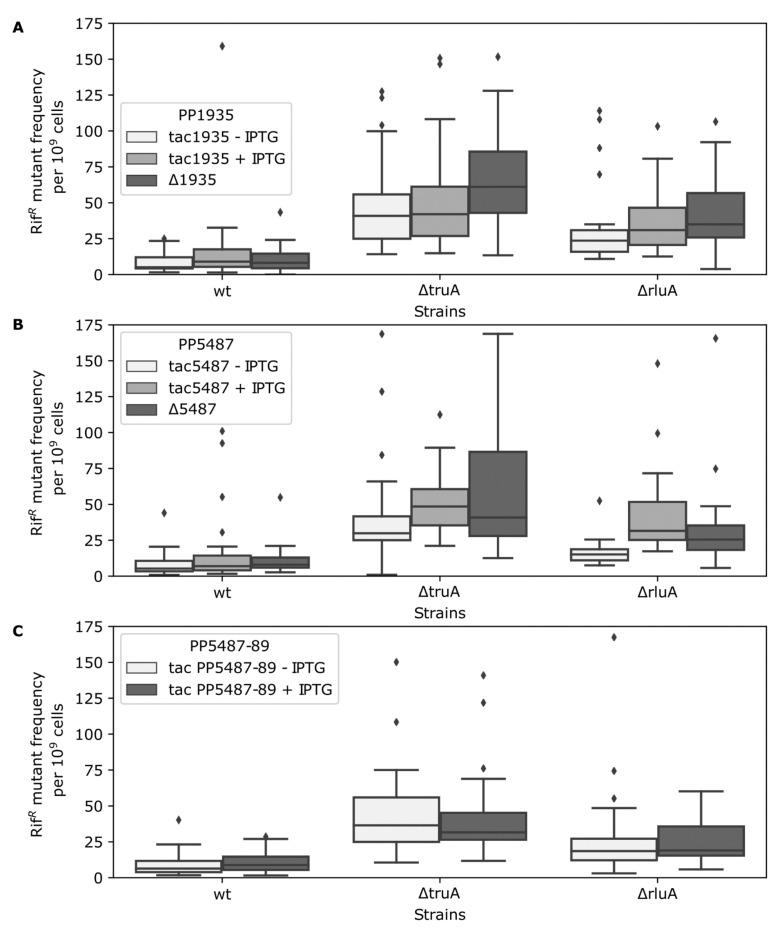
Rif^R^ mutant frequencies of *P. putida* PaW85 (wt) and *truA*- and *rluA-*deficent strains in transcriptional regulator gene *PP1935* and operon *PP5487-89* backgrounds. (**A**) Mutant frequencies in different strains with altered *PP1935* expression. White boxes represent the overexpression of *PP1935* without IPTG, light gray the overexpression with IPTG, and dark grey boxes the deletion of *PP1935*. (**B**) Mutant frequencies in different strains with altered *PP5487* expression. White boxes represent the overexpression of *PP5487* without IPTG, light gray the overexpression with IPTG, and dark grey boxes the deletion of *PP5487*. (**C**) Mutant frequencies in different strains with altered *PP5487-PP5489* operon expression, where white boxes represent the overexpression of *PP5487-PP5489* operon without IPTG and light gray boxes the overexpression with IPTG. The mean values (line in the box) of Rif^R^ mutant frequencies per 10^9^ cells are presented. The upper and lower borders of the box represent third and first quartile, respectively, the whiskers represent non-outlier range and dimonds indicate outliers. In each strain *n* = 30 at least.

Since the transcriptional regulator PP1935 was only detectable in the ΔtruA and ΔrluA strains but not in the wild-type strain, we examined the possibility that the higher expression of PP1935 could be responsible for the downregulation of the operon *PP5487-89* and thereby elevated Rif^R^ mutant frequency in the ΔtruA and ΔrluA strains. If this were the case, the deletion of *PP1935* in the ΔtruA and ΔrluA background should decrease mutant frequency and the overexpression of *PP1935* should increase mutant frequency in the wild-type background. However, our results demonstrated that there is no statistically significant change in the Rif^R^ mutant frequency if the regulator gene *PP1935* is either deleted or overexpressed in the aforementioned strains (Figure 5A, Table S10).

Although the gene of the putative regulator PP1935 is situated right next to the operon *PP5487-89* in the genome of *P. putida*, there remains the possibility that PP1935 does not regulate this operon. Thus, we measured the mutant frequency in *P. putida* cells when the first gene of the operon was either deleted or overexpressed, or the whole operon was overexpressed. The results presented in Figure 5B,C revealed that neither the first gene of the operon (*PP5487*) nor the whole operon (*PP5487-89*) had statistically significant effect on the Rif^R^ mutant frequency (Tables S11 and S12). This pertained to deletion and overexpression of *PP5487* as well as overexpression of the entire *PP5487-89*. Taken together, the elevated Rif^R^ mutant frequency in the absence of TruA or RluA is not the consequence of the downregulation of the operon *PP5487-89*.

## 4. Discussion

In the current study we found that most TruA and RluA target tRNAs in *P. putida* overlap with *E. coli* TruA and RluA targets (Table S2). We also demonstrated that in *P. putida* Asp70 of TruA and Asp57 of RluA are the catalytically crucial residues for the pseudouridylation of uridines at tRNA positions 38–40 or 32, respectively (Figure 1B and Figure S2). Interestingly the absence of TruA- or RluA-dependent pseudouridylation led to the mutator phenotype of *P. putida* (Figure 2A). The lack of TruA elevated mutation frequency also in *P. aeruginosa* (Figure 2B).

Catalytically inactive TruA and RluA of *E. coli* retain substrate binding ability [78,89], which implies that Ψ synthases could have roles beyond pseudouridylation. Indeed, in the case of *E. coli* Ψ synthase TruB it was shown in growth competition experiments that inactivation of tRNA binding had a stronger negative effect on bacterial growth than inactivation of the enzyme’s pseudouridylation activity [90]. Moreover, it was demonstrated that TruB also acts as a tRNA chaperon in addition to catalysing pseudouridylation [91]. We cannot exclude the possibility that TruA and/or RluA could also have some additional role(s) in *P. putida* cells, e.g., by acting on alternative substrate RNAs other than those that have already been described. However, the results presented in Figure 2A indicate that the increased mutation frequency in the TruA- and RluA-deficient strains is associated with the absence of Ψs. Namely, strains expressing TruA and RluA proteins with single mutations of their catalytic Asp residue exhibited the same mutator phenotype as the strains lacking the entire TruA or RluA coding sequences (Figure 2A).

Lack of several tRNA modifications has been shown to affect translation efficiency in a gene-specific way and to cause phenotypes in a multitude of biological processes in budding yeast [92]. Although some tRNA modification enzymes can affect mutation frequency, it does not apply to all these enzymes and to all growth conditions of bacteria. For example, in *E. coli* it was demonstrated that the lack of the dimethylallyltransferase MiaA caused elevated mutation frequency; in the same article, the mutation frequency in a *truA-*deficient mutant (there referred to as *hisT*) was also measured; however, in that case, no difference between the *truA-*deficient mutant and the wild-type strain was detected [93]. The results of our study imply that the effect of the TruA-dependent pseudouridylation on mutation frequency might be wide-spread in *Pseudomonas* species, since the absence of TruA elevated Rif^R^ mutant frequency both in *P. putida* and in *P. aeruginosa* (Figure 2). However, the magnitude of the observed effect on mutagenesis was smaller in *P. aeruginosa* than in *P. putida*, and the mutation frequency was not increased when RluA was missing in *P. aeruginosa* (Figure 2B). Based on in silico analyses the tRNA substrates for RluA in *P. putida* and *P. aeruginosa* are identical and the substrate tRNAs for TruA mostly overlap. However, there are some differences in the overall codon usage between *P. putida* and *P. aeruginosa* (http://www.kazusa.or.jp/codon/), and there might be even greater differences when comparing specific genes in these organisms. One may speculate that such dissimilarities in codon usage could cause different phenotypes of *P. putida* and *P. aeruginosa* TruA- and RluA-deficient strains in mutagenesis assay.

tRNA modifications near the anticodon can affect translation and its fidelity. The modifications at position 34 (the first position of the anticodon) is known to affect base pairing and Wobble interactions [3], and modifications 3’ of the anticodon (especially at position 37) can help maintain the reading frame and to avoid reading frame slippage [2,6]. In *S. enterica* Typhimurium the absence of TruA caused a 50% increase in frameshifting, although the observed effect was specific for one leucine codon studied [6]. On the other hand, in *S. cerevisiae*, the lack of Ψ38 and Ψ39 can reduce stop codon readthrough and +1 frameshifting efficiency [21,22]. Thus, there are controversial results regarding the role of Ψs 38, 39 and 40 in translation fidelity.

It has been shown that some *E. coli* strains with remarkably reduced translation fidelity have elevated mutation frequencies [94,95]. For example, in an *E. coli* strain with editing-defective isoleucine aminoacyl-tRNA synthetase, the spontaneous mutation frequency in exponentially growing bacteria was not increased, but Rif^R^ mutation frequency increased ~15-fold in aging colonies of the same strain [95]. Bacher and Schimmel associated the increased error rate with induction of the SOS response [95]. Mutant glycine tRNA genes (with an anticodon that in addition to glycine can translate aspartate) also elevated mutation frequency and a UV-mutagenesis phenotype that was RecA*-*dependent [94]. Ongoing studies in our laboratory have indicated that depending on codon context, the absence of TruA but not RluA can reduce translation fidelity in *P. putida* (Jürgenstein et al., unpublished data). However, since the effect of the absence of TruA on translation fidelity is rather modest and the lack of RluA has no effect at all, we suggest that reduced translation fidelity is not the main reason for the mutator phenotype observed in *Pseudomonas* strains. However, error frequency is sensitive to the codon context. Therefore, it is possible that the pseudouridines, which bracket the anticodon, affect fidelity of translation of one or a few proteins critical for mutation frequency.

As already mentioned above, in *E. coli* the recorded cases of higher mutation frequency in bacterial strains with reduced translation fidelity have been associated with SOS response and homologous recombination [94,95,96]. However, the results of the current study do not support the involvement of specialized DNA polymerases and SOS response in the mutator phenotype of *P. putida* ΔtruA and ΔrluA strains. This was evidenced by the results demonstrating that deletions of the SOS response-regulated mutagenic *imuABC* operon and the genes encoding specialized polymerases Pol II and Pol IV (*dinB*) did not reduce the Rif^R^ mutant frequency in the TruA- or RluA-deficient *P. putida* strains (Figure 2A and Figure 3B). We also excluded the possibility that malfunctioning of two major DNA repair pathways MMR and NER might have caused elevated mutation frequency in the absence of TruA- or RluA-dependent pseudouridylation of tRNAs (Figure 3A). The malfunction or deficiency of different DNA repair pathway enzymes has been shown to affect molecular spectrum of mutations [97,98], and also the action of some specialized DNA polymerases can affect the spectrum of mutations [99]. However, we did not observe any significant changes in the spectrum of Rif^R^ mutants between the *P. putida* wild-type strain and its TruA- or RluA-deficient derivatives (Table S6). These results also implied that the malfunctioning of the studied DNA repair pathways or induction of specialized DNA polymerases are not the cause of the observed increase in mutation frequency. Moreover, it is important to note here that we did not observe any remarkable changes in the expression of DNA replication, recombination or repair pathway enzymes in the proteomes of ΔtruA and ΔrluA strains (dataset PXD022353).

In different organisms there are many documented phenotypes caused by the lack of TruA or its homologues. In both *S. enterica* Typhimurium [23] and *P. aeruginosa* [24] the lack of *truA* can affect virulence, especially in *S. enterica* Typhimurium where the survival of mice is remarkably higher when infected with a *truA* mutant [23]. In *E. coli* [25] and *S. enterica* Typhimurium [100] the lack of TruA remarkably reduces growth rate. In *S. cerevisiae* the lack of Ψ38–39 causes temperature sensitivity [20,101], and even in human a correlation between homozygotic mutation causing expression of a non-functional Pus3 enzyme (pseudouridylates tRNA positions 38–39) and intellectual disabilities was found [102]. Yet, these results harbour a broad spectrum of phenotypes and it is hard to get the full grasp of TruA’s role(s) and interactions in cells. Also, based on the results of the current study it can be concluded that the lack of TruA causes different phenotypes even in rather closely related bacterial species such as *P. putida* and *P. aeruginosa* (Figure 3D,E). Although the absence of TruA activity elevated mutation frequency both in *P. putida* and *P. aeruginosa* (Figure 2), it appeared that the lack of TruA caused remarkable defects in viability in the presence of different stressors (we used stressors affecting cell wall synthesis, translation or ROS production) in *P. putida,* but in *P. aeruginosa* the same stressors caused only modest effects (Figure 3D,E). As the effect of TruA deficiency on mutation frequency was also more remarkable in *P. putida* than in *P. aeruginosa*, it is possible that the absence of TruA-dependent pseudouridylation influences more biological functions in *P. putida* than in *P. aeruginosa*.

Comparison of the proteomes of the ΔtruA strain and the wild-type strain revealed 18 proteins whose expression level was changed at least two-fold (Figure 4B, Table 1; see also the proteome dataset PXD022353). Among these proteins the abundance of catalase KatE was about three-fold reduced in the ΔtruA strain (Figure 4B, Table 1). In *P. putida* KT2440 there are four different catalases: KatA, KatG [also named KatB in the literature [103]], KatE, and PP2887 [103,104], which can compensate each other. Nevertheless, in the current study the expression level of KatA and KatG was not remarkably changed and PP2887 was not detected in the proteomes of ΔtruA and wild-type strains. The level of expression of the KatE gene has been shown to be higher under various stress conditions of *P. putida*, e.g., in stationary phase, under carbon source limitation, and in the presence of osmotic stress [105]. Hence, the reduced expression of KatE could explain the decreased viability of the ΔtruA strain when exposed to oxidative stress-inducing chemicals (Figure 3D). However, further studies are needed to test this hypothesis.

A notable change in the ΔtruA proteome was the upregulation of HisC and HisD (4.4-fold and 3.8-fold respectively) (Figure 4B, Table 1). In *P. putida* the genes for HisC and HisD are located in the same operon and both enzymes participate in histidine biosynthesis. The expression level of other histidine biosynthesis enzymes whose genes are distributed throughout the genome of *P. putida* was not changed. In *E. coli* and *S. enterica* Typhimurium the histidine biosynthesis genes have been localized in a single operon and the transcription of this operon is regulated by translational attenuator during synthesis of a leader peptide containing 7 consecutive histidines [106]. It has been shown that the lack of TruA modifications in tRNA^his^ causes derepression of the histidine operon in *E. coli* and in *S. enterica* Typhimurium [106,107]. Histidine tRNAs are modified by TruA also in *P. putida* (Table S2). However, DNA sequence analysis of the region locating upstream of the *hisC-hisD* operon in *P. putida* did not reveal any potential histidine rich leader peptide-coding sequence and therefore a mechanism related to elevated HisC and HisD expression in TruA-deficient *P. putida* cells remains speculative. Also, there is no data whether the intracellular amount of histidine is increased as a result of upregulation of HisC and HisD in *P. putida* TruA-deficient strain. However, we wish to note that histidine could play an important role in antioxidative defense. Like many other bacterial species, *P. putida* harbors a histidine degradation pathway [108]. It has been demonstrated for *Pseudomonas fluorescens* that histidine degradation via glutamate provides α-ketoglutarate that neutralizes ROS in an NADPH-independent manner [109]. Intriguingly, histidine was recently reported to also enhance the genotoxicity of hydrogen peroxide in *E. coli* and *P. aeruginosa* [110]. Such opposite effects of histidine on oxidative damage illustrates the complexity of the cellular responses to oxidative stress.

In addition to KatE, the ribosome modulation factor Rmf was also downregulated in the ΔtruA strain (Figure 4B, Table 1). Rmf is involved in the conversion of 70S ribosomes to inactive 100S ribosomes in stationary phase cells to reduce the translation rate [111,112]. In contrast to Rmf, another stress factor CsiD was highly upregulated in the ΔtruA proteome (Table 1, Figure 4B). This carbon starvation-induced protein is strictly regulated by RpoS in *E. coli* [113]. The upregulation of CsiD may indicate a general stress response in cells, although the two other stress-regulated proteins KatE and Rmf were downregulated. However, here it is noteworthy that the expression level of the main stress response regulator RpoS was not changed in the ΔtruA strain. In another proteomic study of *P. putida* it was shown that in stationary phase, among hundreds of other proteins, catalases (KatA, KatG, KatE) and CsiD are strongly upregulated [114].

There are only a few phenotypes of bacteria described when RluA is missing. For example, in *E. coli* wild-type cells outcompeted *rluA* mutant cells under conditions of competitive growth [30]. Our stress tolerance experiments indicate that *P. putida* and *P. aeruginosa rluA* mutant strains tolerate various stressors similarly to the wild-type cells (Figure 3D,E). Moreover, only the lack of the catalytic activity of TruA but not RluA affects mutation frequency in *P. aeruginosa* (Figure 2). Analysis of the proteome of the ΔrluA strain also revealed only minor changes in comparison to the wild-type strain (Table 1 and Figure 4A). We also excluded the possibility that the changes in the expression of the prophage-encoded proteins PP5487-89, or in PP1935 that appeared in the RluA- and TruA-deficient strains (Table 1 and Figure 4) has any role in elevated mutation frequency (Figure 5). Therefore, it is plausible that although mutation frequency is elevated both in the TruA- or RluA-deficient *P. putida* strains, the mechanisms underlying the observed mutator phenotype might not entirely overlap in these strains.

Taking together, our results did not reveal any clear evidence for the involvement of error-prone DNA synthesis or malfunctioning of DNA repair functions in the mutator phenotype in the absence of TruA- or RluA-dependent tRNA pseudouridylation in *P. putida*. Also, our results did not reveal any obvious links between stresses and the mutator phenotypes of the *truA-* or *rluA*-deficient strains. Thus, what other mechanisms are conceivable? One possibility is that the elevated mutation frequency is the outcome of a response to the impairment of some very specific function. In fact, it has been shown in several studies that some tRNA modification enzymes can exert very specific functions. For example, in yeast there are Ψ38 or Ψ39 in at least 19 tRNAs, but the temperature-sensitive phenotype of the Pus3p mutant is mainly due to a defect in tRNA^gln(UUG)^, showing that Ψs can have distinct effects on different tRNAs [101]. In addition, although in *P. aeruginosa* TrmB methylates many tRNAs, the expression of KatA and KatB is selectively upregulated at the translation level, since both, *katA* and *katB* gene, are enriched in Phe and Asp codons [9].

However, we can also not exclude the possibility that the elevated mutation frequency is the sum of small effects on distinct cellular functions, but these effects are too small to be detected individually. Mutation frequency can be elevated under stressful conditions of bacteria due to different reasons, e.g., as a result of increase in the rate of DNA replication errors, which may or may not be triggered by DNA template damage, and/or via impairment of fidelity mechanisms such as proofreading and DNA repair [115]. If all or some of these components contributed only in part to a mutator phenotype, then their individual effects on spontaneous mutation frequency might escape notice. This idea is supported by the results of the analysis of the spectrum of mutations in the *P. putida* TruA- and RluA-deficient strains. Changes in the spectrum of mutations could indicate a specific mechanism (e.g., defects in proofreading of DNA polymerase replication errors, induction of certain error-prone DNA polymerase, malfunctioning of specific DNA repair pathway, *etc*), but in fact, we did not observe any differences in the spectrum of mutations when comparing the wild-type and TruA- or RluA-deficient strains (Table S6). Thus, the lack of pseudouridylation of tRNAs may affect to some extent translation (and thereby functionality) of several proteins which participate in DNA replication and repair or various stress responses. The more the expression or functionality of these proteins is affected, the greater will be the effect on mutation frequency, i.e., the mutator phenotype of *P. putida* TruA- and RluA-deficient strains could be a consequence of cumulative effects on various processes affecting mutation frequency, owning to the lack of pseudouridylation.

## Figures and Tables

**Table 1:** Protein expression levels altered in *Pseudomonas putida* ΔtruA or ΔrluA compared to wild-type PaW85.

Locus	Gene	Protein	Fold Change ^†^	*p*-Value
**ΔtruA vs. wild-type**				
PP_1936		Uncharacterized protein	−10.1	0.00025
PP_5487		Uncharacterized protein	−8.0	6.0 × 10^−6^
PP_5488		Uncharacterized protein	−7.1	1.7 × 10^−6^
PP_0115	*katE*	Catalase	−3.1	0.00025
PP_5700		Uncharacterized protein	−2.8	8.1 × 10^−5^
PP_1789		Haloacid dehalogenase-like family hydrolase	−2.7	6.0 × 10^−7^
PP_5502	*rmf*	Ribosome modulation factor	−2.5	0.0014
PP_2264		Putative Sugar ABC transporter, periplasmic sugar-binding protein	−2.4	1.3 × 10^−5^
PP_1788		Uncharacterized protein	−2.1	0.0003
PP_0238	*ssuD*	Alkanesulfonate monooxygenase	−2.0	0.0024
PP_4496	*rluB*	23S rRNA pseudouridylate synthase B	2.1	9.0 × 10^−5^
PP_0880	*dppC*	Dipeptide ABC transporter-putative membrane subunit	2.2	0.0027
PP_4346	*ddlA*	D-alanine-D-alanine ligase A	2.7	0.00061
PP_0966	*hisD*	Histidinol dehydrogenase	3.8	3.9 × 10^−6^
PP_1025	*leuA*	2-isopropylmalate synthase	4.2	3.2 × 10^−5^
PP_0967	*hisC*	Histidinol-phosphate aminotransferase	4.4	9.5 × 10^−7^
PP_2909	*csiD*	carbon starvation induced protein	5.3	9.4 × 10^−5^
PP_0435		M23/M37 family peptidase.	6.3	0.00048
**ΔrluA vs. wild-type**				
PP_5488		Uncharacterized protein	−5.5	6.0 × 10^−6^
PP_5487		Uncharacterized protein	−5.3	1.9 × 10^−5^
On-off regulated proteins				
Locus	Gene	Protein	Detected in	
**ΔtruA vs. wild-type**				
PP_0170		ABC transporter periplasmic binding protein	wild-type	
PP_2818	*mexD*	multidrug RND transporter MexD	wild-type	
PP_1935		Cro/CI family transcriptional regulator	ΔtruA	
PP_2921		hypothetical protein	ΔtruA	
PP_3601	*garD*	galactarate dehydratase	ΔtruA	
**ΔrluA vs. wild-type**				
PP_4042	zwfB	glucose 6-phosphate 1-dehydrogenase	wild-type	
PP_5162		hypothetical protein	wild-type	
PP_1935		Cro/CI family transcriptional regulator	ΔrluA	

^†^ at least twofold statistically significant changes are presented.

## Supplementary Materials

The following are available online at https://www.mdpi.com/2076-2607/9/1/25/s1, Figure S1: ClustalW alignment of amino acid sequences for TruA and RluA of *P. putida* KT2440 and *E. coli* str. K-12 substrain MG1655. Figure S2: Identification of TruA and RluA pseudouridylation sites in different tRNAs in *P. putida* PaW85 in wild-type (wt) and in *truA* and *rluA* mutants. Figure S3: Comparison of Rif^R^ mutant frequency in *P. putida* PaW85 strains carrying *lacI* P^tac^ gene cassette in their chromosome with (+) and without (−) IPTG. Table S1: Bacterial strains and plasmids used in this study. Table S2: List of *P. putida* KT2440 tRNAs which are predicted to be targets for TruA or RluA. Table S3: Oligonucleotides used in this study. Table S4: Statistical analysis of the results of Rif^R^ mutant frequencies in PaW85 wild-type, TruA-, and RluA-deficient strains, TruA and RluA complementation strains and double mutant ΔΔ. Table S5: Statistical analysis of the results of accumulation of Rif^R^ mutants of PAO1-L strains. Table S6: Percentage of mutations in *rpoB* gene in wild-type *P. putida* and in *truA* and *rluA* mutant sequenced with primer PPrpoB1. Table S7: Statistical analysis of the results of accumulation of Rif^R^ mutants of *P. putida* strains lacking *uvrD*, *uvrDtruA* and *uvrDrluA*. Table S8: Statistical analysis of the results of accumulation of Rif^R^ mutants of *P. putida* strains lacking *imuAB*, *imuC*, *polB* and *dinB* genes (Δpol strain), and in addition *truA* or *rluA* gene. Table S9: Statistical analysis of the results of accumulation of Rif^R^ mutants of strains growing with (+) or without (−) thiourea (TU). Table S10: Statistical analysis of the results of accumulation of Rif^R^ mutants of *P. putida* strains lacking *PP1935* or over expressing *PP1935* (“−“without IPTG and “+” with IPTG) in addition to the deletion of *truA* or *rluA* gene. Table S11: Statistical analysis of the results of accumulation of Rif^R^ mutants of *P. putida* strains lacking *PP5487* or over expressing *PP5487* (“−“without IPTG and “+” with IPTG) in addition to the deletion of *truA* or *rluA* gene. Table S12: Statistical analysis of the results of accumulation of Rif^R^ mutants of *P. putida* strains over expressing operon *PP5487-89* (“−“without IPTG and “+” with IPTG) in addition to the deletion of *truA* or *rluA* gene. Explanatory legend for Supplementary 2.
