# Supplementary material for: Pseudouridines of tRNA Anticodon Stem-Loop Have Unexpected Role in Mutagenesis in Pseudomonas sp
Source: Microorganisms. 2020 Dec 23;9(1):25. doi: 10.3390/microorganisms9010025 (PMC7822408; doi:10.3390/microorganisms9010025)
Supplement: Supplementary file 1 [file microorganisms-09-00025-s001.pdf]

```
CLUSTAL 2.1 multiple sequence alignment


E.coli-TruA     ---------MSDQQQPPVYKIALGIEYDGSKYYGWQRQNE-VRSVQEKLEKALSQVANEP
P.putida-TruA   MLLDIIDTATAESAAEGYSRIALGVEYKGARYRGWQRQASGVPSVQQALEQALSKVANEP
                          ::.      :****:**.*::* ***** . * ***: **:***:*****

E.coli-TruA     ITVFCAGRTDAGVHGTGQVVHFETTALRKDAAWTLGVNANLPGDIAVRWVKTVPDDFHAR
P.putida-TruA   ISVVCAGRTDAGVHGCGQVVHFDTRAVRDERAWTMGTNFNLPHDISVVWSRPMPADFHAR
                *:*.*********** ******:* *:*.: ***:*.* *** **:* * :.:* *****

E.coli-TruA     FSATARRYRYIIYNHRLRPAVLSKGVTHFYEPLDAERMHRAAQCLLGENDFTSFRAVQCQ
P.putida-TruA   FKACARRYRYVIYNDPIRPAHLAEEVTWNHRPLDVDRMAEAAQYLLGTHDFSAFRASQCQ
                *.* ******:***. :*** *:: **  :.***.:** .*** *** :**::*** ***

E.coli-TruA     SRTPWRNVMHINVTRHGPYVVVDIKANAFVHHMVRNIVGSLMEVGAHNQPESWIAELLAA
P.putida-TruA   AKSPIKHIYHLRVTRHGQMIVLDVRATAFLHHMVRNIAGVLMAIGAGERPVAWAREVLEG
                :::* ::: *:.*****  :*:*::*.**:*******.* ** :** ::* :*  *:* .

E.coli-TruA     KDRTLAAATAKAEGLYLVAVDYPDRYDLPKPPMGPLFLAD------
P.putida-TruA   RNRREGGVTAHPYGLYLVQVEYPEAFALPKRYIGPHFLSGYEALAD
                ::*  ...**:. ***** *:**: : ***  :** **:.
```

```
CLUSTAL 2.1 multiple sequence alignment


E.coli-RluA     MGMENYNPPQEPWLVILYQDDHIMVVNKPSGLLSVPGRLEEHKDSVMTRIQR-DYPQAES
P.putida-RluA   --------MPLSNVQILFEDAAILVINKPTLLLSVPGRAEDNKDCLITRLQENGYPDALI
                           . : **::*  *:*:***: ******* *::**.::**:*. .**:*

E.coli-RluA     VHRLDMATSGVIVVALTKAAERELKRQFREREPKKQYVARVWGHPSPAEGLVDLPLICDW
P.putida-RluA   VHRLDWETSGIILLARDADSHRELSRQFHDRETEKAYTALCWGQPALDSGSIDLPLRYDP
                *****  ***:*::*    :.***.***::**.:* *.*  **:*:  .* :****  *

E.coli-RluA     PNRPKQKVCYETGKPAQTEYEVVEYAADNTARVVLKPITGRSHQLRVHMLALGHPILGDR
P.putida-RluA   PTKPRHVVDHEQGKHALTFWRIVERCGD-HCRVELTPITGRSHQLRVHMLSIGHPLLGDR
                *.:*:: * :* ** * * :.:** ..*  .** *.**************::***:****

E.coli-RluA     FYASPEARAMAPRLLLHAEMLTITHPAYGNSMTFKAPADF
P.putida-RluA   LYANPQALAAHERLCLHASMLSFTHPVSGQRLKFECPAPF
                :**.*:* *   ** ***.**::***. *: :.*:.** *
```

Figure S1. ClustalW alignment of amino acid sequences for TruA and RluA of *P. putida* KT2440 and *E. coli* str. K-12 substrain MG1655. Identical amino acids are indicated by asterisks, conserved and semi-conserved amino acids are represented as colon and point, respectively.

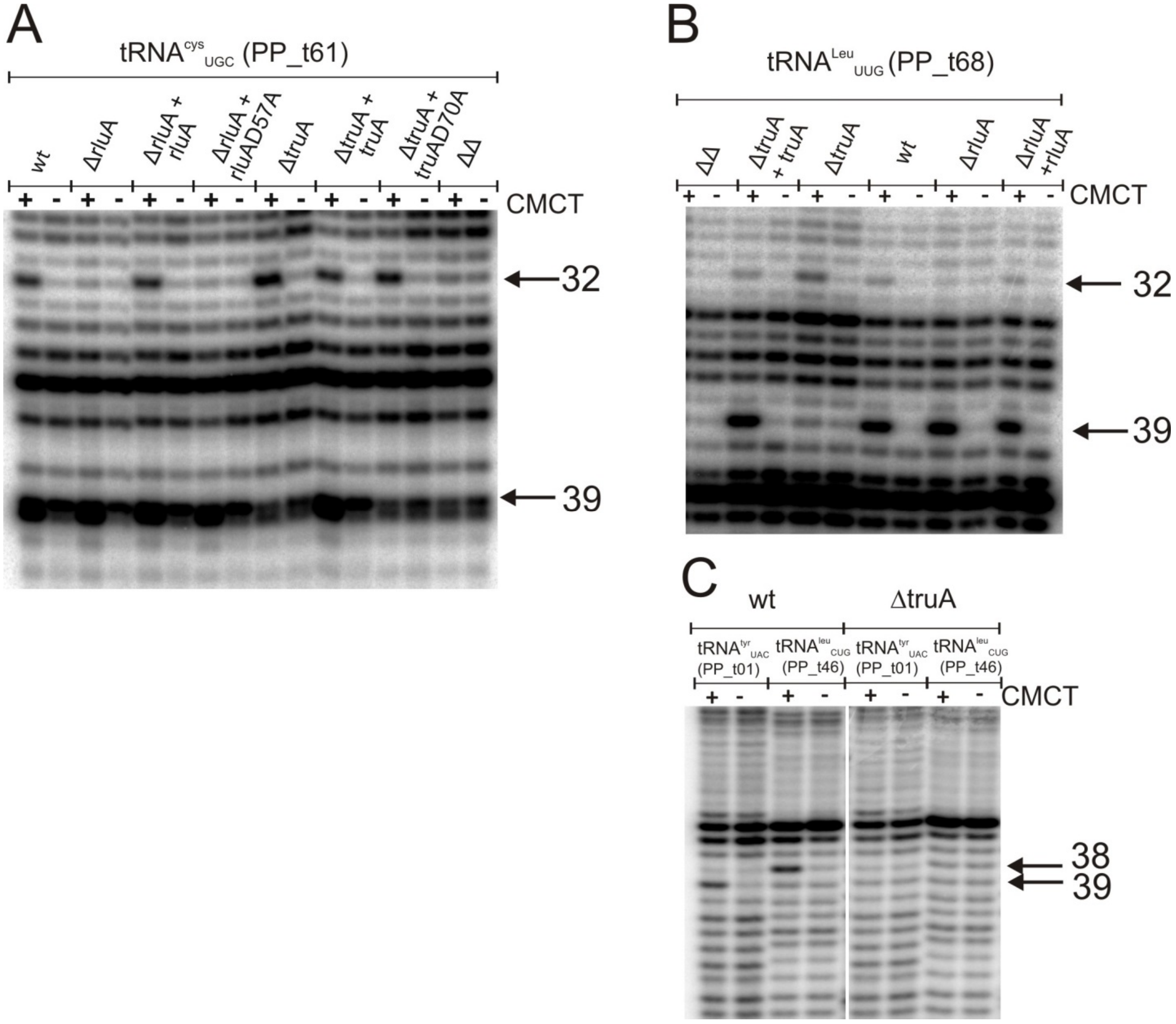

Figure S2. Identification of TruA and RluA pseudouridylation sites in different tRNAs in *P. putida* PaW85 in wild-type (wt) and in *truA* and *rluA* mutants. Analysis of $tRNA^{cys}_{UGC}$ (gene *PP_t61*) which is target for both TruA and RluA (A). Analysis of $tRNA^{leu}_{UUG}$ (gene *PP_t68*) which is target for both TruA and RluA (B). Analysis of $tRNA^{tyr}_{UAG}$ (gene *PP_t01*) and $tRNA^{leu}_{CUG}$ (genes *PP_t46* and *PP_t47*) which are targets for TruA (C). Results of *truA* and *rluA* deletion strains and complementation strains with functional protein (ΔtruA + truA/ΔrluA +rluA) and with catalytically inactive protein (ΔtruA + truA D70A/ΔrluA + rluA D57A) and *truA* and *rluA* double deletion strain (ΔΔ) are presented. "+" corresponds to CMCT treated lane and "-" to untreated tRNA. Ψs are found places where there is CMCT specific stop on "+" lane but not on "-" lane. Arrow with number indicates either the target position of TruA (38 or 39) or RluA (32).

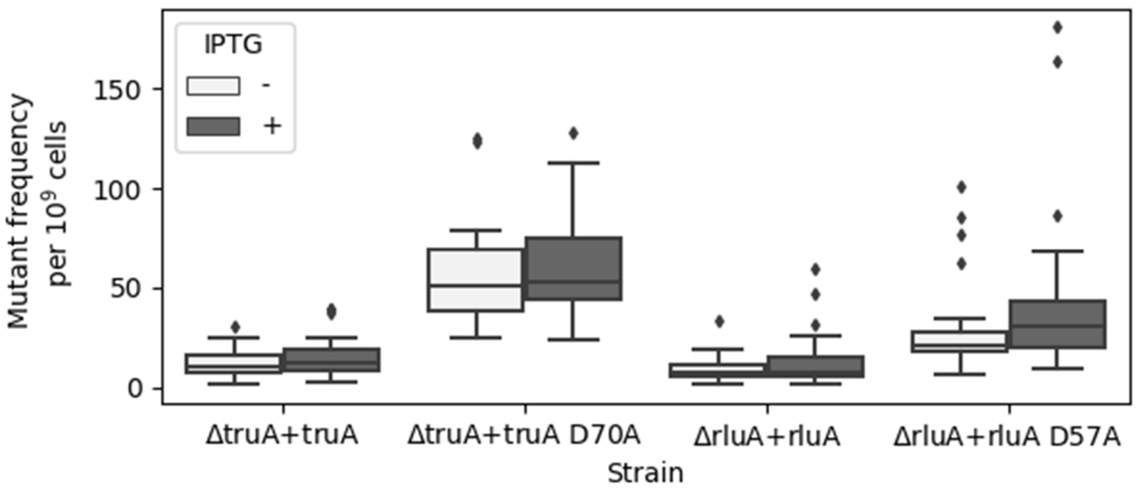

Figure S3. Comparison of Rif^R mutant frequency in *P. putida* PaW85 strains carrying *lacI* P^{tac} gene cassette in their chromosome with (+) and without (-) IPTG. The mean values (line in the box) of Rif^R mutant frequencies per 10^9 cells are presented. The upper and lower boarders of box represent third and first quartile, respectively, the whiskers are non-outlier range and diamonds corresponds for outliers. In each strain n=30.

Table S1. Bacterial strains and plasmids used in this study.

Strain/plasmid	Description	Source
P. putida		
PaW85	Wild type, isogenic to KT2440	[1]
PaWΔtruA	PaW85, Δ*truA* (PP1994)	[2]
PaWΔrluA	PaW85, Δ*rluA* (PP1731)	This study
PaWΔtruAΔrluA	PaW85, Δ*truA* and Δ*rluA*	This study
PaWΔtruA + truA	PaW85, Δ*truA* strain containing *lacI*-P_{tac}-*truA* gene cassette with functional *truA* gene in the intergenic region between *glmS* and *PP5408* (Gm^r)	This study
PaWΔtruA + truAD70A	PaW85, Δ*truA* strain containing *lacI*-P_{tac}-*truA*D70A gene cassette in the intergenic region between *glmS* and *PP5408* (Gm^r). The catalytic aspartic acid of TruA is mutated to alanine.	This study
PaWΔrluA + rluA	PaW85, Δ*rluA* strain containing *lacI*-P_{tac}-*rluA* gene cassette with functional *rluA* gene in the intergenic region between *glmS* and *PP5408* (Gm^r)	This study
PaWΔrluA + rluAD57A	PaW85, Δ*rluA* strain containing *lacI*-P_{tac}-*rluA*D57A gene cassette in the intergenic region between *glmS* and *PP5408* (Gm^r). The catalytic aspartic acid of RluA is mutated to alanine.	This study
PaWΔtruAΔpol	PaW85, Δ*truA,* Δ*imuAB*, Δ*dnaE2*, Δ*polB* and Δ*dinB*	This study
PaWΔrluAΔpol	PaW85, Δ*rluA,* Δ*imuAB*, Δ*dnaE2*, Δ*polB* and Δ*dinB*	This study
PaWΔpol	PaW85, Δ*imuAB*, Δ*dnaE2*, Δ*polB* and Δ*dinB*	This study
PaWΔurvD	PaW85, Δ*urvD*	[3]
PaWΔtruAΔuvrD	Paw85, Δ*truA* and ΔuvrD	This study
PaWΔrluAΔuvrD	Paw85, Δ*rluA* and ΔuvrD	This study
PaWΔPP1935	PaW85, ΔPP*1935*	This study
PaWΔtruAΔPP1935	PaW85, Δ*truA* and ΔPP*1935*	This study
PaWΔrluAΔPP1935	PaW85, Δ*rluA* and ΔPP*1935*	This study
PaWΔPP5487	PaW85, Δ*PP5487*	This study
PaWΔtruAΔPP5487	PaW85, Δ*truA* and Δ*PP5487*	This study
PaWΔrluAΔPP5487	PaW85, Δ*rluA* and Δ*PP5487*	This study
PaW + PP1935	PaW85 containing *lacI*-P_{tac}-*PP1935* gene cassette in the intergenic region between *glmS* and *PP5408* (Gm^r).	This study
PaWΔtruA+ PP1935	PaW85, Δ*truA* strain containing *lacI*-P_{tac}-*PP1935* gene cassette in the intergenic region between *glmS* and *PP5408* (Gm^r).	This study

PaWΔrluA+ PP1935	PaW85, Δ*rluA* strain containing *lacI*-P_{tac}-*PP1935* gene cassette in the intergenic region between *glmS* and *PP5408* (Gm^r).	This study
PaW + PP5487	PaW85 containing *lacI*-P_{tac}-*PP5487* gene cassette in the intergenic region between *glmS* and *PP5408* (Gm^r).	This study
PaWΔtruA+ PP5487	PaW85, Δ*truA* strain containing *lacI*-P_{tac}-PP5487 gene cassette in the intergenic region between *glmS* and *PP5408* (Gm^r).	This study
PaWΔrluA+ PP5487	PaW85, Δ*rluA* strain containing *lacI*-P_{tac}-*PP5487* gene cassette in the intergenic region between *glmS* and *PP5408* (Gm^r).	This study
PaW + opr5487-89	PaW85 containing *lacI*-P_{tac}-*PP5487, PP5488, PP5489* gene cassett in the intergenic region between *glmS* and *PP5408* (Gm^r).	This study
PaWΔtruA+ opr5487-89	PaW85, Δ*truA* strain containing *lacI*-P_{tac}- *PP5487, PP5488, PP5489* gene cassette in the intergenic region between *glmS* and *PP5408* (Gm^r).	This study
PaWΔrluA+ opr5487-89	PaW85, Δ*rluA* strain containing *lacI*-P_{tac}- *PP5487, PP5488, PP5489* gene cassette in the intergenic region between *glmS* and *PP5408* (Gm^r).	This study
E. coli		
DH5α	*supE44* Δ*lacU169 recA1 endA1 hsdR17 thi-1 gyrA96 relA1*	Invitrogen
HB101	*subE44 subF58 hsdS3*(r_B^- m_B^-) *recA13 ara-14 proA2 lacY1 galK2 rpsL20 xyl-5 mtl-1*	[4]
CC118λpir	*Δ(ara-leu) araD* Δ*lacX74 galE galK phoA20 thi-1 rpsE rpoB argE (*Am*) recA1 λpir* phage lysogen	[5]
P. aeruginosa		
PAO1-L	PAO1 subline, University of Lausanne, Dieter Haas collection	Stephan Heeb
PAOΔtruA	PAO1-L, Δ*truA* (PA3114)	This study
PAOΔrluA	PAO1-L, Δ*rluA* (PA3246)	This study
**Plasmids**		
pEMG	Suicide plasmid containing *lacZα* with two flanking I-SceI sites (Km^r)	[6]
pSW (I-SceI)	Plasmid for I-SceI expression (Ap^r)	[7]
pEMG/truA	pEMG with a chimeric PCR insert for deleting *truA* (Km^r)	[2]
pEMG/rluA	pEMG with a chimeric PCR insert for deleting *rluA* (Km^r)	This study
pGP-miniTn7-ΩGm	pGP704 L carrying a SacI–XbaI mini-Tn7-ΩGm cassette from pBK-miniTn7-ΩGm (Ap^r, Gm^r)	[8]
pUX-BF13	Helper plasmid, providing the Tn*7* transposase proteins (Ap^r)	[9]
pBRlacItac	Expression vector containing P_{tac} promoter and lacI repressor in pBR322 (Ap^r)	[10]

pSEVA-Km(RK2)	Cloning vector (Km^r)	[11]
pSEVAHIII/Xba	pSEVA-Km(RK2) with disrupted Hind III and XbaI restriction sites (Km^r)	This study
pSEVA/lacItac	pSEVAHIII/Xba where gene cassette *lacI*-P_{tac} has been inserted as BamHI fragment (Km^r)	This study
pSEVA/lacItactruA	pSEVA/lacItac containing gene cassette *lacI*-P_{tac}-*truA* (Km^r)	This study
pGPTn7/truA	pGP-miniTn7-ΩGm containing gene cassette *lacI*-P_{tac}-*truA* between inverted repeats of mini-Tn*7* (Ap^r, Gm^r)	This study
pSEVA/lacItacrluA	pSEVA/lacItac containing gene cassette *lacI*-P_{tac}-*rluA* (Km^r)	This study
pGPTn7/rluA	pGP-miniTn7-ΩGm containing gene cassette *lacI*-P_{tac}-*rluA* between inverted repeats of mini-Tn*7* (Ap^r, Gm^r)	This study
pBluescriptKS(+)	Cloning vector (Ap^r)	Stratagene
pSEVA/lacItactruA-mut	pSEVA-Km(RK2) containing gene cassette *lacI*-P_{tac}-*truA*D70A where catalytic aspartic acid's codon of TruA has been changed to alanine codon (Km^r)	This study
pGPTn7/truA-mut	pGP-miniTn7-ΩGm containing gene cassette *lacI*-P_{tac}-*truA*D70A between inverted repeats of mini-Tn*7* (Ap^r, Gm^r)	This study
pSEVA/lacItacrluA-mut	pSEVA-Km(RK2) containing gene cassette *lacI*-P_{tac}-*rluA*D57A where catalytic aspartic acid's codon of RluA has been changed to alanine codon (Km^r)	This study
pGPTn7/rluA-mut	pGP-miniTn7-ΩGm containing gene cassette *lacI*-P_{tac}-*rluA*D57A between inverted repeats of mini-Tn*7* (Ap^r, Gm^r)	This study
pEMG/uvrD	pEMG with a chimeric PCR insert for deleting *uvrD* (Km^r)	[3]
pEMG/PP1935	pEMG with a chimeric PCR insert for deleting *PP1935* (Km^r)	This study
pEMG/PP5487	pEMG with a chimeric PCR insert for deleting *PP5487* (Km^r)	This study
pEMG/opr5487-89	pEMG with a chimeric PCR insert for deleting operon *PP5487-PP5489* (Km^r)	This study
pSEVA/lacItacPP1935	pSEVA/lacItac containing gene cassette *lacI*-P_{tac}-*PP1935* (Km^r)	This study
pGPTn7/PP1935	pGP-miniTn7-ΩGm containing gene cassette *lacI*-P_{tac}-*PP1935* between inverted repeats of mini-Tn*7* (Ap^r, Gm^r)	This study
pSEVA/lacItacPP5487	pSEVA/lacItac containing gene cassette *lacI*-P_{tac}-*PP5487* (Km^r)	This study

pGPTn7/PP5487	pGP-miniTn7-ΩGm containing gene cassette *lacI*-P_{tac}-*PP5487* between inverted repeats of mini-Tn*7* (Ap^r, Gm^r)	This study
pSEVA/lacItacopr5487-89	pSEVA/lacItac containing gene cassette *lacI*-P_{tac}-*PP5487*,*PP5488*, and *PP5489* (Km^r)	This study
pGPTn7/opr5487-89	pGP-miniTn7-ΩGm containing gene cassette *lacI*-P_{tac}- *PP5487*, *PP5488*, and *PP5489* between inverted repeats of mini-Tn*7* (Ap^r, Gm^r)	This study

Table S2. List of *P. putida* KT2440 tRNAs which are predicted to be targets for TruA or RluA.

Locus	Amino acid	Anticodon	Position of TruA-targeted U(s)	RluA consensus sequence[†]
PP_t01[‡]	Tyr	GTA	39	
PP_t07, PP_t63	Asn	GTT	39	
PP_t14[‡]	Ser	CGA	39	**U**UCGAAA
PP_t19	Ser	GGA	40	
PP_t25, PP_t27, PP_t29	Ala	GGC	38	
PP_t38, PP_t40	His	GTG	38, 39	
PP_t39	Leu	TAG	38, 39	
PP_t41, PP_t69	Gln	TTG	38	
PP_t42, PP_t43	Val	GAC	38	
PP_t44	Pro	GGG	38	
PP_t45	Leu	TAA	39	
PP_t46, PP_t47[‡]	Leu	CAG	38, 40	
PP_t48	Leu	GAG	38	
PP_t61[‡]	Cys	GCA	39	**U**UGCAAA
PP_t68[‡]	Leu	CAA	39	**U**UCAAAA
PP_t70	Met	CAT	39	
PP_t71	Met	CAT	39	
PP_t72	Phe	GAA	39	**U**UGAAAA
PP_t73	Thr	TGT	39	

[†]the 5´-3´ direction of RluA consensus sequence 5’ ΨUXXAAA 3’ is marked, where U 32 is in bold

[‡]experimentally proved targets of TruA and RluA

Table S3. Oligonucleotides used in this study.

Oligonucleotide	Sequence (5´-3´)[†]	Description/Use
BH-minD	AGGATCCGCCTGCTGGGCAAAGAGAAG	Construction of ΔrluA
RluA-del	GCAGTGCGGCCCTTGCAGGATAGCAGTAAACGCTGG GGC	Construction of ΔrluA
ER-PP1730	AGAATTCTGTAGCTCGGGCTGAGGCTTG	Construction of ΔrluA
RluA-out	TCCTGCAAGGGCCGCACTGC	Construction of ΔrluA
truAalgHII	TATAAGCTTATGCTCTTGGACATCATCGA	For amplification of *truA*
truAloppSal	TATGTCGACTCAGTCTGCCAACGCCTC	For amplification of *truA*
rluAalgHIII	TATAAGCTTATGCCGCTGTCGAATGT	For amplification of *rluA*
rluAloppSal	TATGTCGACTCAGAACGGCGCAGGGCA	For amplification of *rluA*
Eco47-PPtruA	AGGAAGCGCTGGAGCGCGCC	For mutating *truA* catalytic aspartic acid
mut-PPtruAD70A	TGAACGGCAGCGGCGGTACG	For mutating *truA* catalytic aspartic acid
PPrluAsees	CTGCTGCTGTCGGTACCTGG	For mutating *rluA* catalytic aspartic acid
mutPPrluAD57A	GTTTCCCAGGCCAGACGGTG	For mutating *rluA* catalytic aspartic acid
Tn7glmS	AATCTGGCCAAGTCGGTGAC	Verification of Tn*7* insertion
Tn7R109	CAGCATAACTGGACTGATTTCAG	Verification of Tn*7* insertion
pSW-R	AACGTCGTGACTGGGAAAA	Verification of pSW (I-SceI)
pSW-F	GGACGCTTCGCTGAAAACTA	Verification of pSW (I-SceI)
UvrDTS1F	ATAGGTACCGCATGATGACCAAGAGCCCA	Verification of ΔuvrD locus
UvrDTS2Rev	ATATCTAGACGACTGGAAGTGGTTACGGG	Verification of ΔuvrD locus
PP1935eesXba	GGATCTAGACGCTGGCATTTGCCACATACAC	Construction of ΔPP1935
PP1935del	GGGTCGATGGCGCGGTAGAAGTCGAGGCACGAACA GGCAG	Construction of ΔPP1935
PP1935out	TTCTACCGCGCCATCGACCC	Construction of ΔPP1935
PP1935tagaBH	ACGGATCCGAACATGAAAGGTGAGGTCTC	Construction of ΔPP1935
PP5487tagaXba	GGATCTAGAGGAGGCCTGCATGATCGACG	Construction of ΔPP5487
PP5487out	TGAGGAGGTGGTATGTTTCATACTG	Construction of ΔPP5487
PP5487eesBH	ACGGATCCGAGTCCCGCTTCCTCAGCTC	Construction of ΔPP5487 and Δopr5487-89
PP5487del	CAGTATGAAACATACCACCTCCTCAGCGATCCTAATTT TTGGGTGCAG	Construction of ΔPP5487
opr5487tagaXba	CATCTAGACAAGCGATTCAGCTGCTCTGC	Construction of Δopr5487-89
opr5487-89out	GGTAAGCGGTACGTCAGGTTG	Construction of Δopr5487-89
opr5487-89del	CAACCTGACGTACCGCTTACCGCGATCCTAATTTTTGG GTGCAG	Construction of Δopr5487-89
PP5487start-Pst	TACTGCAGATGATAAACGCGAGTGAGTA	Overexpression of *PP5487* and *PP5487-PP5489*
PP5487endXba	CCTCTAGACTATTCTTGCGTTTCGAATTC	Overexpression of *PP5487*

PP5489opr-endXba	ATTCTAGATCAATTGAATTTGTAGGTGTA	Overexpression of *PP5487-PP5489*
PP1935start-Pst	CCCTGCAGATGCTTCCTTTTGGACGCGT	Overexpression of *PP1935*
PP1935stopXba	GGTCTAGACTATTCCGAGTCCCGCTTCCT	Overexpression of *PP1935*
PAOERtruAees	CCGACAACGAATTCACCCTCG	Construction of *P. aeruginosa* ΔtruA
PAOtruAdel	CCTCGGCGAGTGAGCGACGAGGGTCGCTTCATTTCCGG	Construction of *P. aeruginosa* ΔtruA
PArluAout	CCGCCCGCCGATTTCCGGTAC	Construction of *P. aeruginosa* ΔrluA
PArluAtaga-ERI	TAGAATTCCGTTGCGAGCGATCTCCGGG	Construction of *P. aeruginosa* ΔrluA
PprpoB1	GGCGGAAAGCGAAGGCCTG	Sequencing *P. putida rpoB* gene
PprpoB2	CAACGCCTTCTTTCACCACG	Sequencing *P. putida rpoB* gene
PP-leu1	GGAGAAGACTCGAACTTCCACGACC	For primer extension analysis of $tRNA^{leu}_{CUG}$
PP-cys	GCCGAGGTCGGAATCGAACCGGC	For primer extension analysis of $tRNA^{cys}_{UGC}$
PP-ser	GATAGGGATTTGAACCCTAGGTACTG	For primer extension analysis of $tRNA^{ser}_{UCG}$
PP-leu2	CGAGGGAGACTCGAACTCCCACTC	For primer extension analysis of $tRNA^{leu}_{UUC}$
PP-tyr	GATAGGGATTTGAACCCTAGGTACTG	For primer extension analysis of $tRNA^{tyr}_{UAC}$

† The sites for restriction enzymes are underlined.

Table S4. Statistical analysis of the results of Rif^R mutant frequencies in PaW85 wild-type, TruA-, and RluA-deficient strains, TruA and RluA complementation strains and double mutant ΔΔ. p-values of nonparametric Kruskal-Wallis test are presented. Statistically significant values are presented in bold.

	PaW	ΔtruA	ΔtruA +truA	ΔtruA +truAD70A	ΔrluA	ΔrluA +rluA	ΔrluA +rluAD57A	ΔΔ
PaW		**<0.001**	1.0	**<0.001**	**<0.001**	1.0	**0.00028**	**<0.001**
ΔtruA	**<0.001**		**<0.001**	1.0	0.104	**<0.001**	**0.0015**	1.0
ΔtruA+truA	1.0	**<0.001**		**<0.001**	**<0.001**	1.0	0.14	**<0.001**
ΔtruA+truAD70A	**<0.001**	1.0	**<0.001**		0.188	**<0.001**	**0.0034**	1.0
ΔrluA	**<0.001**	0.104	**<0.001**	0.188		**<0.001**	1.0	1.0
ΔrluA+rluA	1.0	**<0.001**	1.0	**<0.001**	**<0.001**		**0.0015**	**<0.001**
ΔrluA+rluAD57A	**<0.001**	**0.0015**	0.138	**0.0034**	1.0	**0.0015**		**<0.001**
ΔΔ	**<0.001**	1.0	**<0.001**	1.0	1.0	**<0.001**	**0.025**	

Table S5. Statistical analysis of the results of accumulation of Rif[R] mutants of PAO1-L strains. p-values of nonparametric Kruskal-Wallis test are presented. Statistically significant values are presented in bold.

	PAO	PAO ΔtruA	PAO ΔrluA	PAO ΔΔ
PAO		**<0.001**	0.0826	**<0.001**
PAO ΔtruA	**<0.001**		**<0.001**	1.0
PAO ΔrluA	0.862	**<0.001**		**0.001**
PAO ΔΔ	**<0.001**	1.0	**0.001**	

Table S6. Percentage of mutations in *rpoB* gene in wild-type *P. putida* and in *truA* and *rluA* mutant sequenced with primer PPrpoB1.

Position in *rpoB* gene	Mutation	wt†	ΔtruA	ΔrluA
1546	C - G	0.6	0	0
1550	C - T	1.2	1.56	4.84
1552	C - A	1.2	0	0
1553	A - G	8.38	10.94	6.45
1553	A - T	11.38	9.38	6.45
1553	A - C	1.2	0	1.61
1561	G - T	1.2	0	0
1561	G - A	0.6	0	0
1562	A - G	37.72	43.75	37.10
1569	del CAA	0.6	0	0
1580	C - T	2.99	1.56	4.84
1591	C - T	3.59	4.69	12.90
1591	C - G	0.6	0	0
1592	A - T	3.59	6.25	0
1592	A - G	10.78	18.75	17.74
1607	C - T	9.58	1.56	6.45
1612	C - A	4.79	0	0
1706	C - T	0	1.56	1.61
Total sequenced mutants		167	64	62

†Results from [12]

Table S7. Statistical analysis of the results of accumulation of Rif^R mutants of *P. putida* strains lacking *uvrD*, *uvrDtruA* and *uvrDrluA*. p-values of nonparametric Kruskal-Wallis test are presented. Statistically significant values are presented in bold.

	ΔuvrD	ΔuvrDΔtruA	ΔuvrDΔrluA
ΔuvrD		**<0.001**	**<0.001**
ΔuvrDΔtruA	**<0.001**		**0.03**
ΔuvrDΔrluA	**<0.001**	**0.03**	

Table S8. Statistical analysis of the results of accumulation of Rif^R mutants of *P. putida* strains lacking *imuAB*, *imuC*, *polB* and *dinB* genes (Δpol strain), and in addition *truA* or *rluA* gene. p-values of nonparametric Kruskal-Wallis test are presented. Statistically significant values are presented in bold.

	wt	ΔpolΔtruA	ΔpolΔrluA	Δpol
wt		**<0.001**	**<0.001**	0.95
ΔpolΔtruA	**<0.001**		1	**<0.001**
ΔpolΔrluA	**<0.001**	1		**<0.001**
Δpol	0.95	**<0.001**	**<0.001**	

Table S9. Statistical analysis of the results of accumulation of Rif^R mutants of strains growing with (+) or without (-) thiourea (TU). p-values of nonparametric Kruskal-Wallis test are presented. Statistically significant values are presented in bold.

	wt-	wt+	ΔtruA-	ΔtruA+	ΔrluA-	ΔrluA+
wt-		1.0	**<0.001**	**<0.001**	**<0.001**	**<0.001**
wt+	1.0		**<0.001**	**<0.001**	**<0.001**	**0.006**
ΔtruA-	**<0.001**	**<0.001**		1.0	1.0	1.0
ΔtruA+	**<0.001**	**<0.001**	1.0		0.69	1.0
ΔrluA-	**<0.001**	**<0.001**	1.0	0.69		1.0
ΔrluA+	**<0.001**	**0.006**	1.0	1.0	1.0	

Table S10. Statistical analysis of the results of accumulation of Rif^R mutants of *P. putida* strains lacking *PP1935* or over expressing *PP1935* ("- "without IPTG and "+" with IPTG) in addition to the deletion of *truA* or *rluA* gene. p-values of nonparametric Kruskal-Wallis test are presented. Statistically significant values are presented in bold.

	tac 1935-	tac 1935+	Δ1935	ΔtruA tac1935-	ΔtruA tac1935+	ΔtruA Δ1935	ΔrluA tac1935-	ΔrluA tac1935+	ΔrluA Δ1935
tac1935-		1.0	1.0	**<0.001**	**<0.001**	**<0.001**	**0.002**	**<0.001**	**<0.001**
tac1935+	1.0		1.0	**<0.001**	**<0.001**	**<0.001**	0.08	**<0.001**	**<0.001**
Δ1935	1.0	1.0		**<0.001**	**<0.001**	**<0.001**	**0.013**	**<0.001**	**<0.001**
ΔtruAtac1935-	**<0.001**	**<0.001**	**<0.001**		1.0	1.0	1.0	1.0	1.0
ΔtruAtac1935+	**<0.001**	**<0.001**	**<0.001**	1.0		1.0	0.8	1.0	1.0
ΔtruAΔ1935	**<0.001**	**<0.001**	**<0.001**	1.0	1.0		**0.006**	0.49	1.0
ΔrluAtac1935-	**0.002**	0.08	**0.0013**	1.0	0.8	**0.006**		1.0	1.0
ΔrluAtac1935+	**<0.001**	**<0.001**	**<0.001**	1.0	1.0	0.49	1.0		1.0
ΔrluAΔ1935	**<0.001**	**<0.001**	**<0.001**	1.0	1.0	1.0	1.0	1.0	

Table S11. Statistical analysis of the results of accumulation of Rif^R mutants of *P. putida* strains lacking *PP5487* or over expressing *PP5487* ("- "without IPTG and "+" with IPTG) in addition to the deletion of *truA* or *rluA* gene. p-values of nonparametric Kruskal-Wallis test are presented. Statistically significant values are presented in bold.

	tac 5487-	tac 5487+	Δ5487	ΔtruA tac5487-	ΔtruA tac5487+	ΔtruA Δ5487	ΔrluA tac5487-	ΔrluA tac5487+	ΔrluA Δ5487
tac5487-		1.0	1.0	**<0.001**	**<0.001**	**<0.001**	0.29	**<0.001**	**<0.001**
tac5487+	1.0		1.0	**<0.001**	**<0.001**	**<0.001**	1.0	**<0.001**	**0.006**
Δ5487	1.0	1.0		**<0.001**	**<0.001**	**<0.001**	1.0	**<0.001**	**0.001**
ΔtruAtac5487-	**<0.001**	**<0.001**	**<0.001**		1.0	1.0	**0.01**	1.0	1.0
ΔtruAtac5487+	**<0.001**	**<0.001**	**<0.001**	1.0		1.0	**<0.001**	1.0	0.36
ΔtruAΔ5487	**<0.001**	**<0.001**	**<0.001**	1.0	1.0		**<0.001**	1.0	0.9
ΔrluAtac5487-	0.29	1.0	1.0	**0.01**	**<0.001**	**<0.001**		**0.002**	0.29
ΔrluAtac5487+	**<0.001**	**<0.001**	**<0.001**	1.0	1.0	1.0	**0.002**		1.0
ΔrluAΔ5487	**<0.001**	**0.006**	**0.001**	1.0	0.36	0.9	0.29	1.0	

Table S12. Statistical analysis of the results of accumulation of Rif^R mutants of *P. putida* strains over expressing operon *PP5487-89* ("- "without IPTG and "+" with IPTG) in addition to the deletion of *truA* or *rluA* gene. p-values of nonparametric Kruskal-Wallis test are presented. Statistically significant values are presented in bold.

	tac5487-89-	tac5487-89+	ΔtruA tac5487-89-	ΔtruA tac5487-89+	ΔrluA tac5487-89-	ΔrluA tac5487-89+
tac5487-89-		1.0	**<0.001**	**<0.001**	**0.003**	**<0.001**
tac5487-89+	1.0		**<0.001**	**<0.001**	0.06	**0.002**
ΔtruA tac5487-89-	**<0.001**	**<0.001**		1.0	**0.007**	0.19
ΔtruA tac5487-89+	**<0.001**	**<0.001**	1.0		0.054	0.87
ΔrluA tac5487-89-	**0.003**	0.06	**0.007**	0.054		1.0
ΔrluA tac5487-89+	**<0.001**	**0.002**	0.19	0.87	1.0	

Explanatory legend for Supplementary 2. The data used for proteome analysis of *P. putida* strains (wt, *ΔtruA*, *ΔrluA*) is available via ProteomeXchange with identifier PXD022353. The dataset includes spreadsheet designated as Supplementary 2. This spreadsheet has 3 sheets, Sheet1 contains explanation for Sheet2 and Sheet3. Sheets 2 and 3 contain a list of proteins identified in analysis of differences in protein abundance between *P. putida* wild-type and *truA* or *rluA* deletion strain respectively. On sheets 2 and 3 columns A-C ("Gene name" to "Protein names") have been obtained from UniProtKB database, columns D-L ("LFQ intensity (log2) wt1" to "Sequence coverage [%]") are the corresponding experimental data obtained from the label-free quantification of whole cell proteome and columns M-R ("Student's T-test Significant"-"Fold change") contain the statistical analysis of the proteome data, performed with Perseus software.

References

1. Bayley SA, Duggleby CJ, Worsey MJ, Williams PA, Hardy KG, Broda P. Two modes of loss of the Tol function from *Pseudomonas putida* mt-2. Mol Gen Genet. 1977;154(2):203-4.
2. Tagel M, Tavita K, Horak R, Kivisaar M, Ilves H. A novel papillation assay for the identification of genes affecting mutation rate in *Pseudomonas putida* and other pseudomonads. Mutat Res. 2016;790:41-55. doi: 10.1016/j.mrfmmm.2016.06.002.
3. Ukkivi K, Kivisaar M. Involvement of transcription-coupled repair factor Mfd and DNA helicase UvrD in mutational processes in *Pseudomonas putida*. DNA Repair (Amst). 2018;72:18-27. doi: 10.1016/j.dnarep.2018.09.011.
4. Boyer HW, Roulland-Dussoix D. A complementation analysis of the restriction and modification of DNA in *Escherichia coli*. J Mol Biol. 1969;41(3):459-72.
5. de Lorenzo V, Herrero M, Jakubzik U, Timmis KN. Mini-Tn*5* transposon derivatives for insertion mutagenesis, promoter probing, and chromosomal insertion of cloned DNA in gram-negative eubacteria. J Bacteriol. 1990;172(11):6568-72.
6. Martinez-Garcia E, de Lorenzo V. Engineering multiple genomic deletions in Gram-negative bacteria: analysis of the multi-resistant antibiotic profile of *Pseudomonas putida* KT2440. Environ Microbiol. 2011;13(10):2702-16. doi: 10.1111/j.1462-2920.2011.02538.x.
7. Wong SM, Mekalanos JJ. Genetic footprinting with mariner-based transposition in *Pseudomonas aeruginosa*. Proc Natl Acad Sci U S A. 2000;97(18):10191-6.
8. Jakovleva J, Teppo A, Velts A, Saumaa S, Moor H, Kivisaar M, et al. Fis regulates the competitiveness of *Pseudomonas putida* on barley roots by inducing biofilm formation. Microbiology. 2012;158(Pt 3):708-20. doi: 10.1099/mic.0.053355-0.
9. Bao Y, Lies DP, Fu H, Roberts GP. An improved Tn*7*-based system for the single-copy insertion of cloned genes into chromosomes of gram-negative bacteria. Gene. 1991;109(1):167-8.
10. Ojangu EL, Tover A, Teras R, Kivisaar M. Effects of combination of different -10 hexamers and downstream sequences on stationary-phase-specific sigma factor sigma(S)-dependent transcription in *Pseudomonas putida*. J Bacteriol. 2000;182(23):6707-13.
11. Silva-Rocha R, Martinez-Garcia E, Calles B, Chavarria M, Arce-Rodriguez A, de Las Heras A, et al. The Standard European Vector Architecture (SEVA): a coherent platform for the analysis and deployment of complex prokaryotic phenotypes. Nucleic Acids Res. 2013;41(Database issue):D666-75. doi: 10.1093/nar/gks1119.
12. Jatsenko T, Tover A, Tegova R, Kivisaar M. Molecular characterization of Rif(r) mutations in *Pseudomonas aeruginosa* and *Pseudomonas putida*. Mutat Res. 2010;683(1-2):106-14. doi: 10.1016/j.mrfmmm.2009.10.015.
